# Novel Crizotinib–GnRH Conjugates Revealed the Significance of Lysosomal Trapping in GnRH-Based Drug Delivery Systems

**DOI:** 10.3390/ijms20225590

**Published:** 2019-11-08

**Authors:** József Murányi, Attila Varga, Pál Gyulavári, Kinga Pénzes, Csilla E. Németh, Miklós Csala, Lilla Pethő, Antal Csámpai, Gábor Halmos, István Peták, István Vályi-Nagy

**Affiliations:** 1 MTA-SE Pathobiochemistry Research Group, Tűzoltó St. 37-47, H1094 Budapest, Hungary; varga.attila@med.semmelweis-univ.hu (A.V.); gyulavari.pal@med.semmelweis-univ.hu (P.G.); penzes.kinga@med.semmelweis-univ.hu (K.P.); 2 Department of Medical Chemistry, Molecular Biology and Pathobiochemistry, Semmelweis University, H1094 Budapest, Hungary; nemeth.csilla@med.semmelweis-univ.hu (C.E.N.); csala.miklos@med.semmelweis-univ.hu (M.C.); 3 MTA-ELTE Research Group of Peptide Chemistry, Eötvös Loránd University, H1117 Budapest, Hungary; 4 Institute of Chemistry, Eötvös Loránd University, H1117 Budapest, Hungary; csampai@chem.elte.hu; 5 Department of Biopharmacy, Faculty of Pharmacy, University of Debrecen, H4032 Debrecen, Hungary; halmos.gabor@pharm.unideb.hu; 6 Oncompass Medicine Hungary Ltd., H1024 Budapest, Hungary; istvan.petak@oncompassmedicine.com; 7 Central Hospital of Southern Pest National Institute of Hematology and Infectious Diseases, H1097 Budapest, Hungary; drvnistvan@gmail.com

**Keywords:** targeted drug delivery 1, GnRH 2, GnRHR 3, lysosome 3, permeability 4, crizotinib 5, conjugate 6, NSCLC 7, c-Met 8, endocytosis 9, galectin 10

## Abstract

Several promising anti-cancer drug–GnRH (gonadotropin-releasing hormone) conjugates have been developed in the last two decades, although none of them have been approved for clinical use yet. Crizotinib is an effective multi-target kinase inhibitor, approved against anaplastic lymphoma kinase (ALK)- or ROS proto-oncogene 1 (ROS-1)-positive non-small cell lung carcinoma (NSCLC); however, its application is accompanied by serious side effects. In order to deliver crizotinib selectively into the tumor cells, we synthesized novel crizotinib analogues and conjugated them to a [d-Lys^6^]–GnRH-I targeting peptide. Our most prominent crizotinib–GnRH conjugates, the amide-bond-containing [d-Lys^6^(crizotinib*)]–GnRH-I and the ester-bond-containing [d-Lys^6^(MJ55*)]–GnRH-I, were able to bind to GnRH-receptor (GnRHR) and exert a potent c-Met kinase inhibitory effect. The efficacy of compounds was tested on the *MET*-amplified and GnRHR-expressing EBC-1 NSCLC cells. In vitro pharmacological profiling led to the conclusion that that crizotinib–GnRH conjugates are transported directly into lysosomes, where the membrane permeability of crizotinib is diminished. As a consequence of GnRHR-mediated endocytosis, GnRH-conjugated crizotinib bypasses its molecular targets—the ATP-binding site of RTKs— and is sequestered in the lysosomes. These results explained the lower efficacy of crizotinib–GnRH conjugates in EBC-1 cells, and led to the conclusion that drug escape from the lysosomes is a major challenge in the development of clinically relevant anti-cancer drug–GnRH conjugates.

## 1. Introduction

Gonadotropin-releasing hormone (GnRH) is a tropic hormone which plays a crucial role in the regulation of the pituitary–gonadal axis. The receptor of GnRH (GnRHR) is expressed mainly in the pituitary gonadotroph cells, but its moderate expression has also been identified in certain extrapituitary tissues, such as ovarium, endometrium, prostate, or lymphocytes [1].

It is well known that several types of cancer cells maintain an elevated GnRHR expression, pointing to the role of GnRH/GnRHR system in the development of these tumors. Furthermore, this phenomenon creates an opportunity for targeted drug delivery. Direct anti-tumor effects, which have been reported using both GnRH agonists and antagonist analogues, represent a further advantage of GnRH analogues as anti-cancer drug delivery peptides [2].

In the last two decades, several GnRH analogues have been developed for targeted drug delivery [3]. It is generally accepted that synthetic GnRH analogues are able to deliver the conjugated drug into GnRHR-expressing cancer cells. Many promising in vitro and in vivo results have been reported that support GnRH-targeted drug delivery [4,5,6,7]. Despite all these initial achievements, none of these anti-cancer drug–GnRH conjugates have so far been approved for clinical use. Additionally, the intracellular transport mechanism of GnRH-based drug delivery systems has not been well investigated. 

Signal transduction therapy is one of the most intensively developing fields of cancer treatment. Approved kinase inhibitors have several advantages compared to classical chemotherapeutic drugs. However, their limited selectivity, the accompanying serious side effects, and the fast-developing resistance against these small-molecule inhibitors still represent a set of general problems that must be addressed [8].

c-Met (hepatocyte growth factor receptor) tyrosine kinase is a remarkable target in signal transduction therapy of cancer, due to its relatively frequent overexpression and the associated poor prognosis. Deregulated activity of c-Met has been reported in breast, colon, lung, pancreas, liver, and ovarian cancers [9]. One of the most serious problems with the inhibition of c-Met is that it plays a vital role in physiological processes such as embryogenesis, wound healing, and organ regeneration [10].

Crizotinib is a first-generation tyrosine kinase inhibitor initially designed to target c-Met [11], of which the (R)-enantiomer has been approved for the treatment of ALK (anaplastic lymphoma kinase)- and ROS-1 (ROS proto-oncogene 1)-positive advanced NSCLC (non-small cell lung carcinoma) as a multi-target inhibitor. In association with its potent c-Met inhibitory effect, crizotinib causes several serious side effects that limit its therapeutic applications and significantly impair patients’ quality of life [12]. Nevertheless, application of crizotinib as a c-Met inhibitor is still being intensively investigated, and it is under clinical trial in several types of advanced cancers (https://clinicaltrials.gov). Interestingly, the (S)-enantiomer of crizotinib differs in pharmacological profile from the (R)-enantiomer, but it also bears promising therapeutic potential for cancer therapy, [13,14].

According to the literature data on anti-cancer GnRH conjugates, we hypothesized that the GnRHR-targeted delivery of crizotinib might reduce side effects, improve efficacy, and even delay the development of resistance [6]. In addition, we supposed that the target-specific delivery could extend the applicability of both the (R)- and (S)-enantiomers of crizotinib. Moreover, the enhanced selectivity of crizotinib might allow a synergistic and tolerable combination with other kinase inhibitors (e.g., with inhibitors of epidermal growth factor receptor (EGFR) [15]).

After consideration of the data outlined above, we designed racemic crizotinib analogues in order to synthesize novel crizotinib–GnRH conjugates for further development. Contrary to our initial goals, biological evaluation of crizotinib–GnRH conjugates revealed a serious impediment of GnRH-based drug delivery, which had not previously been identified. Lysosomal trapping mechanisms of small-molecule kinase inhibitors [16] and nanoparticle-based protein delivery systems [17] have come into the spotlight recently. Here, we demonstrate clear evidence of the lysosomal sequestration of GnRHR-targeted crizotinib–GnRH conjugates, a phenomenon which should be taken into account in the development of anti-cancer GnRH conjugates with therapeutic value.

## 2. Results

### 2.1. Design of Crizotinib–GNRH Conjugates

**Figure 1:**
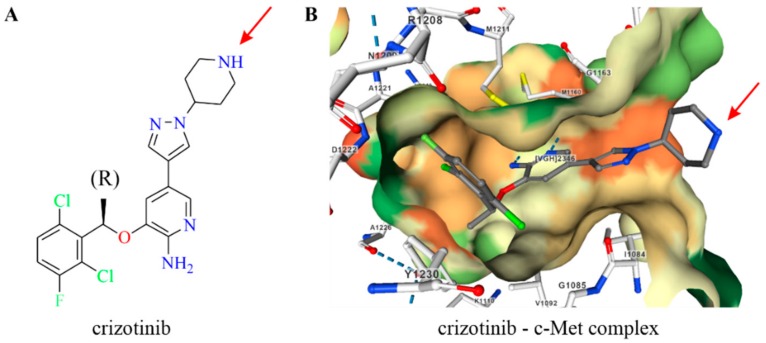
The nitrogen atom of piperidine in crizotinib was selected for conjugation (marked by red arrow). (**A**) Structure of (R)-crizotinib; (**B**) X-ray structure of crizotinib bound to the kinase domain of c-Met (2WGJ). The hydrophobicity of the ATP-binding pocket is colored from red (hydrophilic) to green (hydrophobic). Hydrogen bonds are marked with blue dashed lines.

The cocrystal structure of the c-Met-crizotinib complex revealed that the 2-aminopyridine core of crizotinib plays a crucial role in binding to the ATP-binding pocket of ALK and c-Met kinases. On the other hand, the piperidine ring of crizotinib is directed to the solvent, serving as a sterically accessible site for conjugation to targeting agents (Figure 1).

### 2.2. Synthesis of Crizotinib Analogues and Crizotinib–GNRH Conjugates

**Figure 2:**
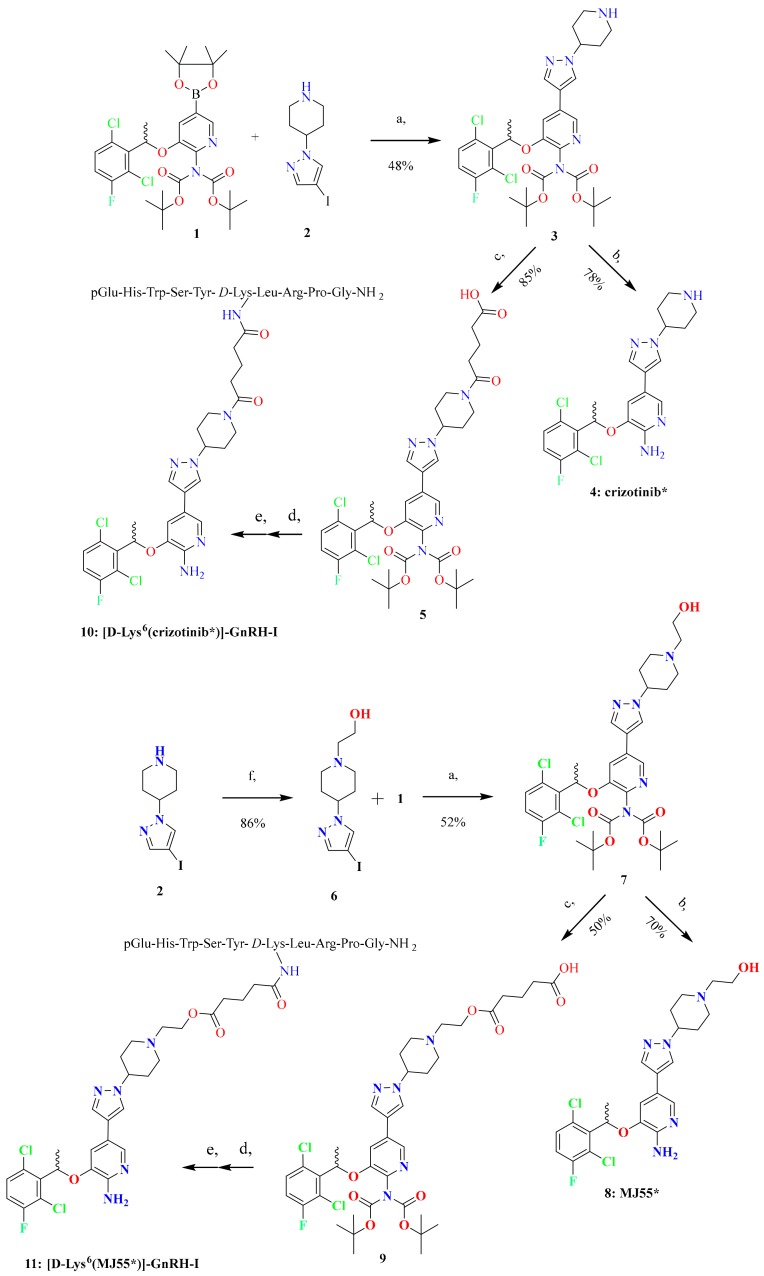
Synthesis of crizotinib*, [d-Lys^6^(crizotinib*)]–GnRH-I, MJ55*, and [d-Lys^6^(MJ55*)]–GnRH-I. (**a**) 2 M Cs_2_CO_3_ in H_2_O, Pd(dppf)Cl_2_*, dimethyl sulfoxide, 70 °C, 3 h; (**b**) trifluoroacetic acid/water (9:1 ratio) 25 °C, 2 h; (**c**) glutaric anhydride, triethylamine, CH_2_Cl_2_, 25 °C, 2 h; (**d**) COMU**, 4-methylmorpholine, *N,N*-dimethylformamide, 25 °C, 2 h; (**e**) trifluoroacetic acid /phenol/water/triisopropyl silane (88:5:5:2 ratio) 25 °C, 2 h; (**f**) K_2_CO_3_, bromoethanol, tetrahydrofuran, reflux, 24 h. *Pd(dppf)Cl_2_—[1,1′-Bis(diphenylphosphino)ferrocene]dichloropalladium(II). **COMU—(1-Cyano-2-ethoxy-2-oxoethylidenaminooxy)dimethylamino-morpholino-carbenium hexafluorophosphate.

The synthetic routes to racemic crizotinib (compound **4**, marked as crizotinib*), racemic MJ55 (compound **8**, marked as MJ55*), [d-Lys^6^(crizotinib*)]–GnRH-I (compound **10**), and [d-Lys^6^(MJ55*)]–GnRH-I (compound **11**) are shown in Figure 2. 

We selected the nitrogen atom of piperidine in crizotinib for modification, in order to keep the 2-aminopyridine core intact, which is responsible for the inhibition of receptor tyrosine kinases (RTKs). Thus, the 2-aminopyridine core was protected by two tert-butyloxycarbonyl-protecting (BOC) groups in the boronic acid pinacol ester **1**. Suzuki coupling of **1** with iodopyrazoles **2** and **6** afforded intermediates **3** and **7**, respectively. The acid-catalysed deprotection of **3** and **7** led to crizotinib* and its novel hydroxyethyl derivative MJ55*, respectively. 

Regioselective acylation of **3** with glutaric anhydride gave **5**, where an amide bond was formed between crizotinib* and the glutaric acid spacer. The carboxyl group of **5** was then coupled to the ε-amino group of [d-Lys^6^]–GnRH-I on resin. Finally, the conjugate was cleaved from the resin, purified by RP-HPLC, and lyophilized, resulting in the formation of [d-Lys^6^(crizotinib*)]–GnRH-I. Employing the same reaction sequence, hydroxyethyl derivative **7** was also converted into the corresponding conjugate [d-Lys^6^(MJ55*)]–GnRH-I, in which the glutaric acid spacer develops forms an ester bond with the conjugated MJ55*.

### 2.3. GnRHR Expression and GnRH Uptake of EBC-1 NSCLC Cells

**Figure 3:**
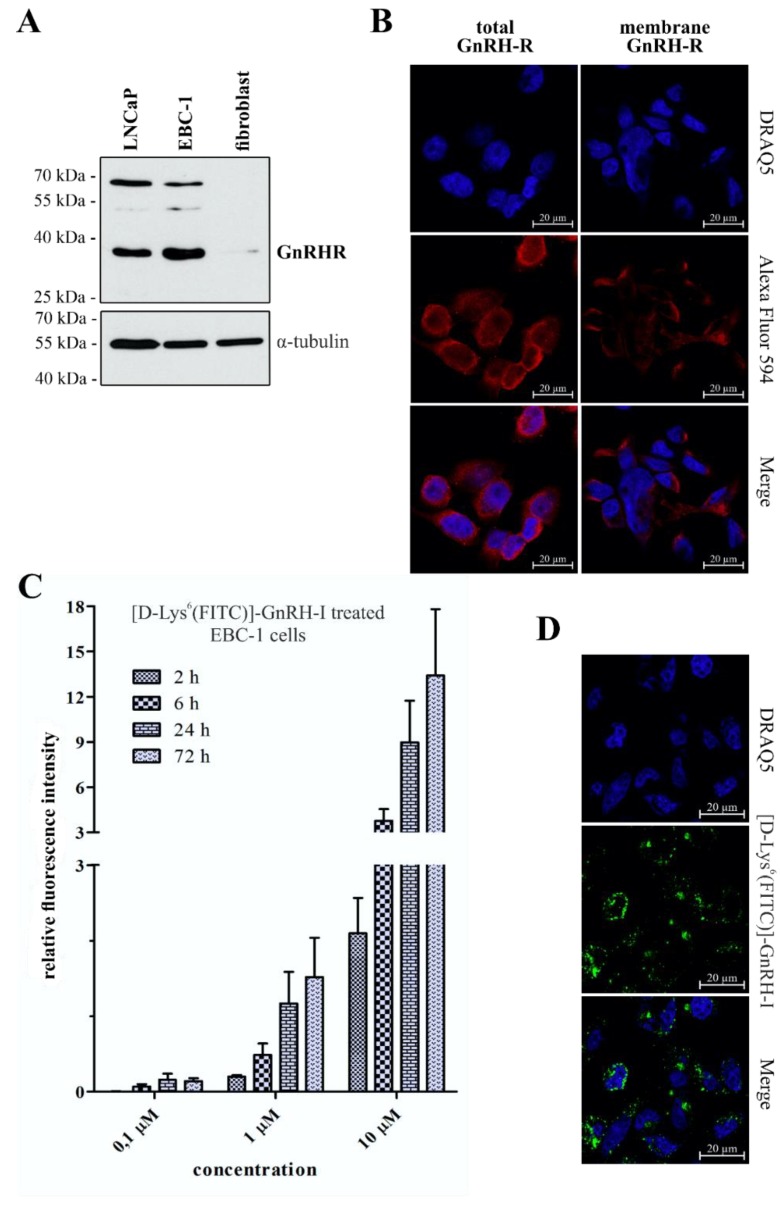
GnRHR expression and cellular uptake of [d-Lys^6^(FITC)]–GnRH-I on EBC-1 cells. (**A**) Expression of GnRHR in EBC-1 cells, confirmed by western blot; (**B**) EBC-1 cells contain a high level of GnRHR (total GnRHR) and a high proportion of these receptors are located in the plasma membrane (membrane GnRHR). Nuclei: blue (DRAQ5), GnRHR: red (Alexa Fluor 594); (**C**) Time- and concentration-dependent dynamic uptake of [d-Lys^6^(FITC)]–GnRH-I was confirmed by flow cytometry. Results are presented as mean relative fluorescent intensity ± SD (*N* = 3); (**D**) Intracellular localization of [d-Lys^6^(FITC)]–GnRH-I (green; 10 µM, 24 h) in EBC-1 cells was confirmed by confocal microscopy (nuclei: blue).

The *MET*-amplified non-small cell lung cancer (NSCLC) cell line EBC-1 was selected as an in vitro model to investigate the efficacy of the novel crizotinib–GnRH conjugates. The GnRHR expression of EBC-1 cells was confirmed by western blot, as shown in Figure 3A. GnRHR-expressing LNCaP prostate cancer cells [18] were used as a positive control, and primary human skin fibroblast cells were selected as a negative control. 

As shown in Figure 3B, confocal laser scanning microscopy (CLSM) experiments underlined that EBC-1 cells express high levels of GnRHR, and also exhibit a significant number of receptors on their plasma membrane. CLSM images of GnRHR negative primary fibroblast cells are available in the Supplementary Information (Section S3, Figure S5).

On EBC-1 cells, the cellular uptake of GnRH-I was measured by flow cytometry. For this method, fluorescein isothiocyanate (FITC)-labeled [d-Lys^6^]–GnRH-I was used. Synthesis and characterisation of [d-Lys^6^(FITC)]–GnRH-I has been described previously [18]. As is shown in Figure 3C, EBC-1 cells exhibited concentration-dependent dynamic uptake of [d-Lys^6^(FITC)]–GnRH-I. The CLSM images shown in Figure 3D further confirmed the intracellular accumulation of [d-Lys^6^(FITC)]–GnRH-I (10 µM, 24 h).

### 2.4. Viability Inhibition Efficacy of Compounds on EBC-1 Cells and Primary Skin Fibroblast Cells

**Figure 4:**
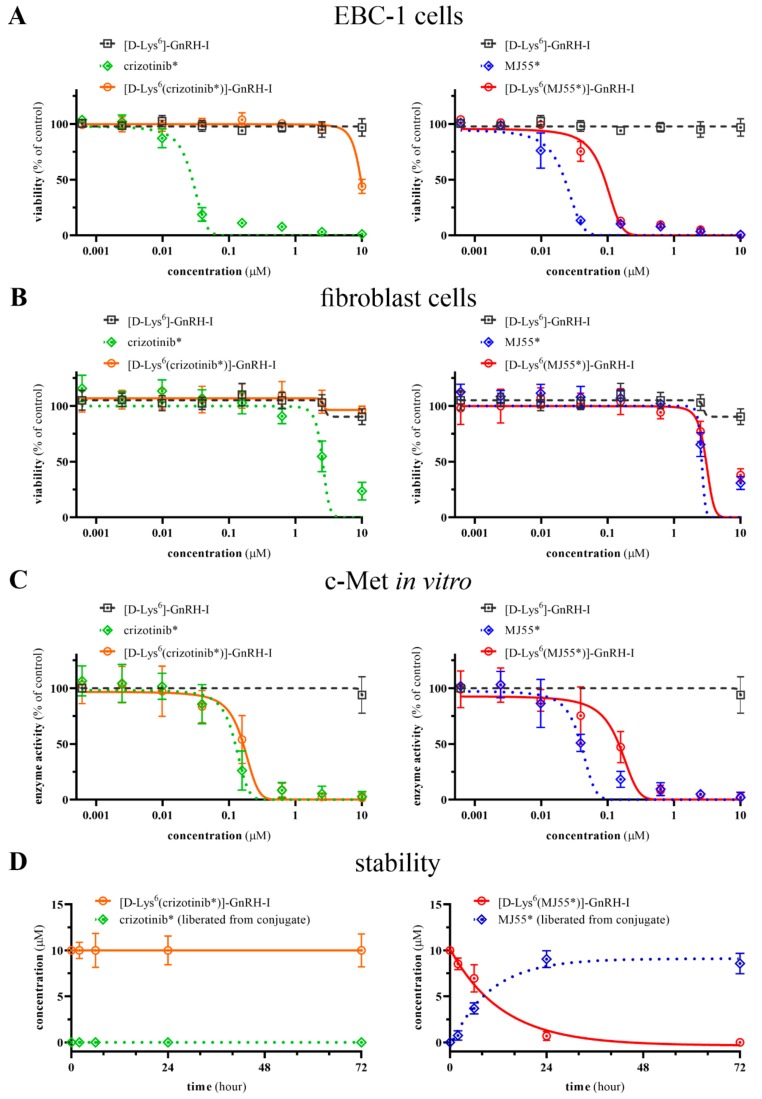
Biological evaluation of compounds. (**A**) Viability inhibition effect on EBC-1 NSCLC cells; (**B**) viability inhibition effect on human primary skin fibroblast cells; (**C**) in vitro inhibition effect on the recombinant c-Met kinase; (**D**) stability in cell culture medium at 37 °C; All values are the mean ± SD of at least three independent experiments.

Viability inhibition potency of crizotinib*, [d-Lys^6^(crizotinib*)]–GnRH-I, MJ55*, [d-Lys^6^(MJ55*)]–GnRH-I, and [d-Lys^6^]–GnRH-I was investigated in EBC-1 NSCLC cells and human primary skin fibroblast cells by CellTiter-Glo assay (Figure 4A). While crizotinib* exerted a potent viability inhibition effect on EBC-1 cells (IC_50_ = 28 nM), the [d-Lys^6^(crizotinib*)]–GnRH-I conjugate did not show any significant effect at the submicromolar range, similarly to the vehicle [d-Lys^6^]–GnRH-I. In contrast, the novel crizotinib analogue (MJ55*) resulted in a similar effect to crizotinib* (IC_50_ = 23 nM), and the ester-bond-containing [d-Lys^6^(MJ55*)]–GnRH-I conjugate also proved to be effective on the EBC-1 cells (IC_50_ = 90 nM).

The adverse cell-toxic effect of these novel compounds was measured on the GnRHR-negative primary human skin fibroblast cells, using the same method as for the EBC-1 cells. As can be seen in Figure 4B, crizotinib* (IC_50_ = 2.6 µM), MJ55* (IC_50_ = 2.6 µM), and [d-Lys^6^(MJ55*)]–GnRH-I (IC_50_ = 3.0 µM) were less effective on healthy fibroblast cells than on EBC-1 cancer cells. However, the non-target-specific cytotoxic effect of [d-Lys^6^(MJ55*)]–GnRH-I was not reduced significantly compared to the free MJ55*, despite the lack of GnRHR on the fibroblast cells.

### 2.5. In Vitro c-Met Inhibition Efficacy of Compounds

c-Met inhibitory effect was investigated using an in vitro recombinant kinase assay. As shown in Figure 4C, MJ55* resulted in potent c-Met inhibition (IC_50_ = 39 nM) and was more effective than crizotinib* (IC_50_ = 123 nM). As was expected (Figure 1), the GnRH-conjugated crizotinib* in [d-Lys^6^(crizotinib*)]–GnRH-I strongly inhibited the c-Met kinase (IC_50_ = 163 nM) as well. Although the [d-Lys^6^(MJ55*)]–GnRH-I conjugate resulted in potent c-Met inhibition (IC_50_ ~ 147 nM), its exact IC_50_ value could not be determined due to the released free MJ55* (detailed in Section 2.6). The vehicle [d-Lys^6^]–GnRH-I did not inhibit c-Met kinase, even at the highest concentration (10 µM).

### 2.6. Stability of Compounds in Cell Culture Medium

Stability of compounds was investigated in Eagle’s minimum essential medium (EMEM) cell culture medium at 37 °C. As shown in Figure 4D, [d-Lys^6^]–GnRH-I, crizotinib*, MJ55*, and the amide-bond-containing [d-Lys^6^(crizotinib*)]–GnRH-I conjugate were proven to be stable. Their degradation was not significant even after 72 h. However, free MJ55* released from the ester-bond-containing [d-Lys^6^(MJ55*)]–GnRH-I conjugate was clearly detectable after just 2 h of incubation. The half-life of the ester bond is approximately 8 h. HPLC-UV chromatograms are available in the Supplementary Information (Section S1, Figures S2 and S3).

### 2.7. GnRHR-Binding Affinity of Conjugates

The binding potency of crizotinib–GnRH conjugates to human pituitary GnRHR and prostate cancer GnRHR was investigated by a ligand competition assay. Displacement of [^125^I]-[d-Trp^6^]–GnRH-I as radioligand by the unlabeled crizotinib–GnRH conjugates as competitors was determined. As shown in Table 1, crizotinib–GnRH conjugates could effectively bind to both types of GnRHR at submicromolar concentrations.

### 2.8. c-Met Inhibition of Compounds in EBC-1 Cells

**Figure 5:**
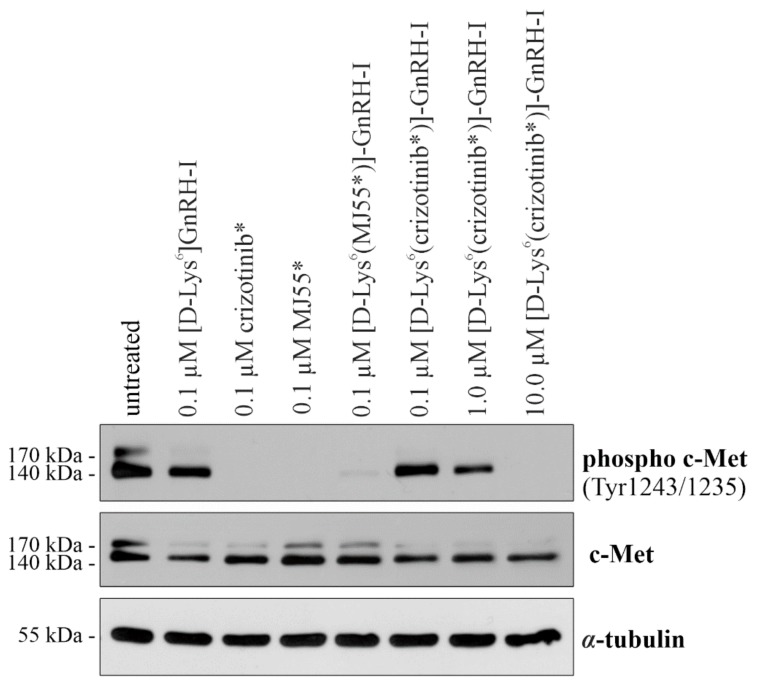
The c-Met inbibitory effect of the compounds in EBC-1 cells. Cells were treated with [d-Lys^6^]–GnRH-I, crizotinib*, MJ55*, [d-Lys^6^(crizotinib*)]–GnRH-I, and [d-Lys^6^(MJ55*)]–GnRH-I for 6 h, and c-Met phosphorylation was analyzed by western blot. Untreated cells and the [d-Lys^6^]–GnRH-I-treated cells were used as negative controls. α-tubulin was used as the loading control (*N* = 3).

As shown in Figure 5, both crizotinib* and MJ55* completely inhibited the phosphorylation of c-Met at 100 nM after 6 h. Similarly to these free drugs, the unstable [d-Lys^6^(MJ55*)]–GnRH-I conjugate also blocked c-Met phosphorylation at 100 nM. However, the stable [d-Lys^6^(crizotinib*)]–GnRH-I had no significant inhibitory effect at the same concentration. Its effect was moderate at 1 µM, and the phosphorylation of c-Met was abolished only at 10 µM. The vehicle [d-Lys^6^]–GnRH-I did not inhibit the c-Met kinase.

### 2.9. Colocalization of [d-Lys^6^(FITC)]–GnRH-I and Lysosomes

**Figure 6:**
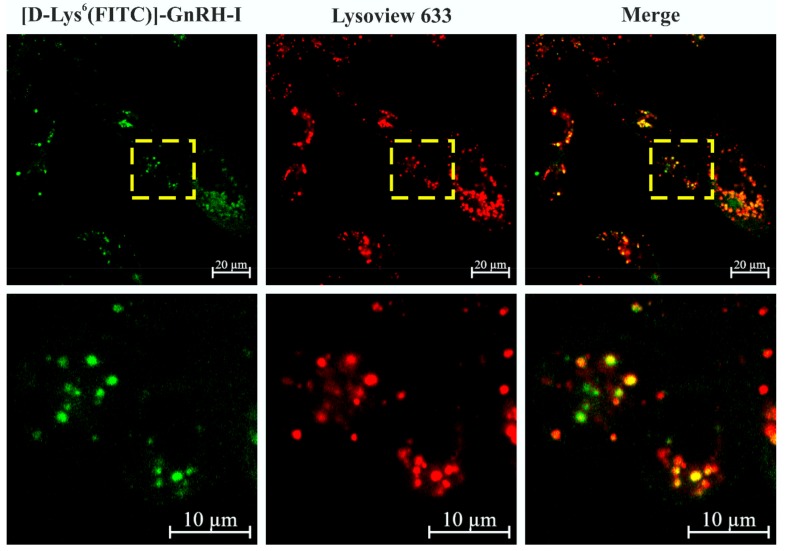
Colocalization of [d-Lys^6^(FITC)]–GnRH-I and the lysosomes in EBC-1 cells. The green fluorescent signal of [d-Lys^6^(FITC)]–GnRH-I (10 µM, 24 h) showed strong colocalization with the red fluorescent signal of lysosome marker Lysoview 633.

To investigate the intacellular localization of [d-Lys^6^]–GnRH-I, we treated the EBC-1 cells with 10 µM [d-Lys^6^(FITC)]–GnRH-I for 24 h, followed by staining with Lysoview 633 fluorescent dye. As shown in Figure 6, the localization of FITC-labeled [d-Lys^6^]–GnRH-I highly correlated with the signal of lysosome marker Lysoview 633. To validate the colocalization, we excluded the possibility of a “cross-talk” effect between the two fluorescent dyes (Section S3, Figure S6).

### 2.10. The pH-Dependent Permeability of Crizotinib* and MJ55*

**Figure 7:**
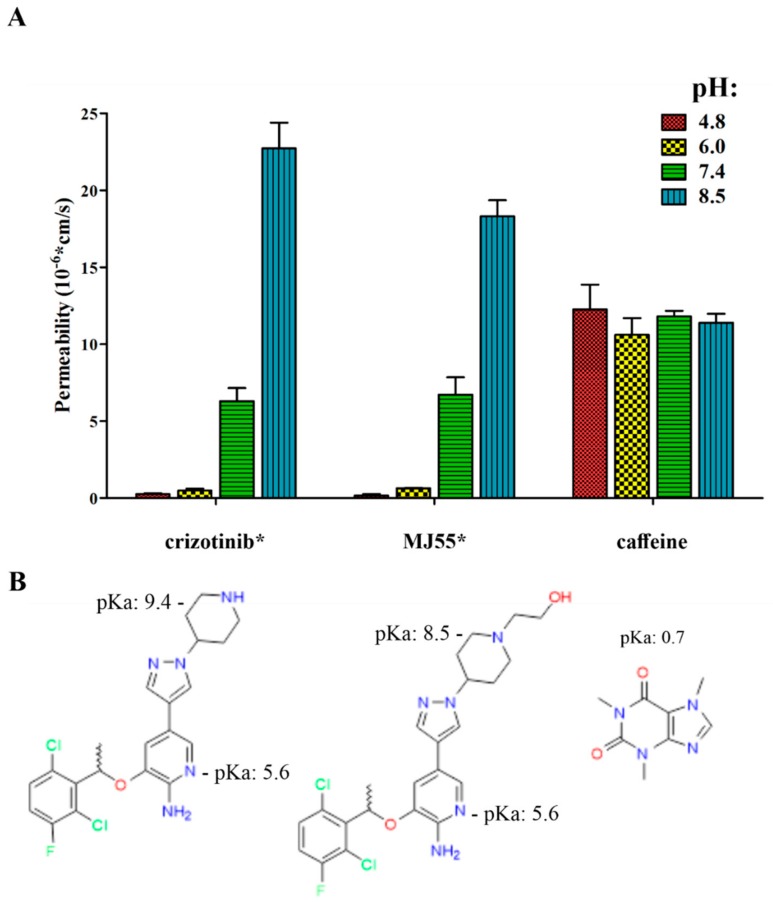
The pH-dependent artificial membrane permeability assay revealed the lysosomotropic character of crizotinib* and MJ55*. (**A**) Permeability of crizotinib* and MJ55*. Caffeine was used as control; (**B**) the pKa value of the pyridinium cation is ~5.6 in crizotinib [20] and MJ55*. The pKa value of the piperidinium cation is 9.4 in crizotinib* [20] and 8.5 in MJ55* (predicted by ChemDraw Prime 15.1). The pKa value of the caffeine cation is 0.7 (https://pubchem.ncbi.nlm.nih.gov/compound/caffeine).

As shown in Figure 7, pH-dependent artificial membrane permeability assay (PAMPA) revealed that the permeability of crizotinib* and MJ55* decreased at lower pH due to the ionization of their 2-aminopyridine core (pKa of the pyridinium cation is ~5.6 [20]). The permeability of crizotinib* and MJ55* was near zero at the early endosome-/lysosome-like pH (pH = 6.0 and 4.8). On the other hand, the permeability of the free drugs increased at mildly basic pH (due to the reduced ionization of the piperidine), which is responsible for their enhanced uptake from the gastrointestinal tract when they are orally administered. Caffeine was used as the positive control. Caffeine remained neutral at the investigated pH range, and its permeability therefore remained constant. The [d-Lys^6^(crizotinib*)]–GnRH-I conjugate turned out not to be membrane-permeable at pH 4.8, 6.0, and 7.4 (data not shown).

### 2.11. [d-Lys^6^(crizotinib*)]–GnRH-I Resulted in Concentration-Dependent Lysosomal Membrane Permeabilization

**Figure 8:**
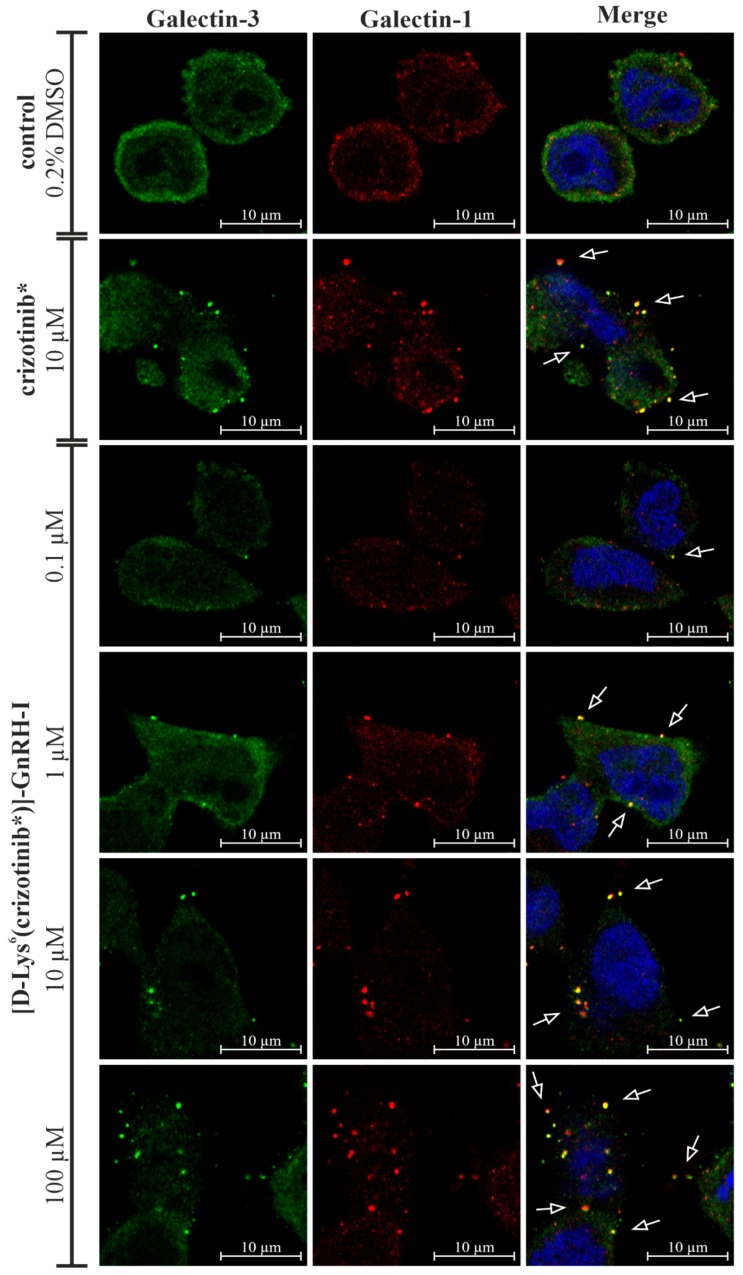
Galectin puncta assay for crizotinib* and [d-Lys^6^(crizotinib*)]–GnRH-I in EBC-1 cells. Galectin-3 and galectin-1 puncta formation indicate lysosomal membrane damage. Galectin-3: green (Alexa Fluor 488); galectin-1: red (Alexa Fluor 594); nuclei: blue (DRAQ5).

Lectin galactoside binding soluble (galectin) puncta assay was used to study lysosomal membrane permeabilization (LMP) effect by crizotinib* and [d-Lys^6^(crizotinib*)]–GnRH-I in EBC-1 cells. Both galectin-1 and galectin-3 were immunocytochemically labeled to investigate their cololaization and puncta formation. CLSM imaging revealed a diffuse distribution of galectin-3 and galectin-1 in untreated cells without puncta formation. Strong galectin-3 and galectin-1 puncta formation was detected in 10 µM crizotinib*-treated cells. The colocalization of galectin-1 and galectin-3 punctas refers to the disruptive lysosomal membrane damage. In the case of [d-Lys^6^(crizotinib*)]–GnRH-I treated cells, puncta formation was negligable at 0.1 µM. However, puncta formation was clearly detectable at 1 µM, and it became more and more significant at 10 µM and at 100 µM (Figure 8).

## 3. Discussion

GnRH-based drug delivery systems are currently being intensively investigated and are a promising field in cancer therapy. Despite the encouraging preclinical results on GnRH-targeted anti-cancer drugs, none of the studied GnRH conjugates have been approved for clinical use yet. The limited success of GnRH conjugates indicates an insufficient understanding of the GnRH/GnRHR systems in cancers and the possible drawbacks of GnRHR targeting, which had not been highlighted previously. For example, one of the most prominent conjugates, zoptarelin doxorubicin (AEZS-108, AN-152), entered into a clinical trial based on its promising preclinical results [21,22]. However, a phase III clinical trial on endometrial cancer concluded that zoptarelin doxorubicin could neither extend overall survival, nor improve the safety profile compared to doxorubicin. Therefore, clinical studies of zoptarelin doxorubicin were discontinued in May 2017 (https://clinicaltrials.gov). During the biological evaluation of our novel crizotinib–GnRH conjugates, we kept the identification of the possible limitations in focus, in order to identify new approaches in the field of GnRH-based drug delivery.

According to the literature data on the structure of crizotinib–protein complexes (http://www.rcsb.org/), we rationally designed and synthesized racemic crizotinib analogues that maintained their RTK-binding affinity, but could also be coupled to targeting moieties, such as [d-Lys^6^]–GnRH-I peptide. In order to keep the GnRHR-binding ability of [d-Lys^6^]–GnRH-I, we applied a glutaric acid spacer between the peptide and the targeted drug. The assumed suitability of glutaric acid as a spacer was supported by several publications on GnRH conjugates [4,23].

In order to perform a regioselective reaction of glutaric anhydride with the nitrogen atom of piperidine in crizotinib, the 2-amino group of pyridine ring must be protected. Upon consideration, we found that the selectively BOC-protected crizotinib racemate might be an appropriate compound to synthesize our first conjugate, the amide-bond-containing [d-Lys^6^(crizotinib*)]–GnRH-I. Literature data on GnRH conjugates represent several possible functional groups able to attach the drug with the spacer. Among these solutions, ester linkages have been widely utilized, since the attached drug can be liberated by hydrolysis of the ester bond [24]. In order to allow ester linkages between the drug and the spacer, we designed and synthesized several hydroxyl-group-containing crizotinib analogues. Using these analogues, we also synthesized several GnRH conjugates. From these compounds, based on their IC_50_ values in EBC-1 cells, only the most potent one, MJ55* and its conjugate, [d-Lys^6^(MJ55*)]–GnRH-I, are detailed in this article.

To investigate the efficacy of our compounds, we first focused on their c-Met inhibitory potential. Accordingly, the *MET*-amplified EBC-1 NSCLC cell line was selected as an in vitro model. Since lung cancers have been less investigated in the context of GnRH targeting, we proved the high GnRHR expression of EBC-1 cells by western blot for the first time. CLSM images demonstrated that a significant proportion of GnRHR is localized into the plasma membrane of EBC-1 cells. This is essential information, because the restrained translocation and the relatively low amount of human GnRHR on the plasma membrane of cancer cells are common phenomena which could limit effective GnRHR targeting [25,26,27]. Finally, the time- and concentration-dependent uptake of FITC-labeled [d-Lys^6^]–GnRH-I was demonstrated by flow cytometry. In brief, our findings on the EBC-1 lung cancer cells correlate with those publications that have highlighted that lung cancer might be an effective target for GnRH-based drug delivery [28,29].

After we proved that EBC-1 is a suitable in vitro model, we tested the viability inhibition efficacy of our compounds on this cell line. The novel MJ55* crizotinib analogue and the ester-bond-containing [d-Lys^6^(MJ55*)]–GnRH-I resulted in high effects similar to crizotinib*. However, the amide-bond-containing [d-Lys^6^(crizotinib*)]–GnRH-I resulted in only a moderate effect at the highest 10 µM concentration. The molecular background of this unexpected result had been investigated further.

The non-target-specific cytotoxic effect of the compounds was measured in the GnRHR-negative primary human skin fibroblast cells. The viability inhibition effect of free crizotinib* and MJ55* on healthy primary fibroblast cells was much weaker than on EBC-1 cancer cells, proving their therapeutic relevance. Surprisingly, the [d-Lys^6^(MJ55*)]–GnRH-I conjugate resulted in a similar cytotoxic effect to the free MJ55*, despite the lack of GnRHR on fibroblast cells. To resolve this contradiction, we determined the stability of the compounds in EMEM cell culture medium under the same conditions that were used for the CellTiter-Glo viability assay. All of the tested compounds proved to be stable, except the ester-bond-containing [d-Lys^6^(MJ55*)]–GnRH-I conjugate. The [d-Lys^6^(MJ55*)]–GnRH-I had a half-life of 8 h in EMEM, due to the hydrolysis of the ester bond, which process releases the free MJ55*.

The early-released free MJ55* (released before its conjugated form was taken up by GnRHR-mediated endocytosis) ensured the high efficacy of [d-Lys^6^(MJ55*)]–GnRH-I on the EBC-1 cells, but it was also responsible for the cytotoxic effect of this compound on the GnRHR-negative primary fibroblast cells. In a similar way, the poor enzymatic stability of the ester bond can also be held responsible for the sustained side effects of zoptarelin doxorubicin. The half-life of zoptarelin doxorubicin is approximately 2 h in human serum [30]. These findings indicate that the application of ester bonds should be considered in targeted drug delivery systems, and the positive preliminary results should be handled with caution because of the possible selectivity problems.

Regarding the stable [d-Lys^6^(crizotinib*)]–GnRH-I conjugate, our main question was why GnRHR-targeting abrogated the efficacy of crizotinib* on EBC-1 cells. To answer this question, we investigated the binding affinity of our conjugates to the human pituitary GnRHR and prostate cancer GnRHR. The displacement analyses of radiolabeled triptorelin ([^125^I]-[d-Trp^6^]–GnRH-I) by unlabeled crizotinib–GnRH conjugates confirmed our hypothesis that the glutaric acid spacer on the d-Lys^6^ of GnRH-I preserved the receptor-binding affinity of both conjugates to both types of GnRHR. Thereafter, results obtained from the recombinant c-Met kinase assay underlined that the piperidine ring is a suitable site for conjugation in crizotinib*, and that the attached targeting structure does not impair the binding affinity to the c-Met kinase.

These results suggest that conjugated crizotinib* was unable reach its intracellular target, the ATP-binding site of c-Met. To strengthen this hypothesis, we examined the activity of c-Met in EBC-1 cells. In correlation with the results of the cell viability assay, crizotinib*, MJ55*, and the unstable [d-Lys^6^(MJ55*)]–GnRH-I diminished the phosphorylation of c-Met at 100 nM. Nevertheless, the stable [d-Lys^6^(crizotinib*)]–GnRH-I proved that GnRH-conjugated crizotinib could not inhibit the phosphorylation of c-Met in EBC-1 cells at submicromolar concentration. 

Next, we investigated the intracellular localization of GnRH-I, using [d-Lys^6^(FITC)]–GnRH-I and a previously described method [18]. According to the literature data on GnRHR-mediated endocytosis [31,32], we counterstained the lysosomes as the suspected destination for the ligands of GnRHR. CLSM images strengthened that [d-Lys^6^(FITC)]–GnRH-I is concentrated in the lysosomes (Figure 6), with similar implications for [d-Lys^6^(crizotinib*)]–GnRH-I. In addition, the artificial membrane permeability experiment revealed the lysosomotropic properties of the 2-aminopyridine core in crizotinib* and MJ55*, as well (Figure 7). Lysosomal accumulation of free crizotinib was also reported by Da Silva et al. in 2015 [33]. 

**Figure 9:**
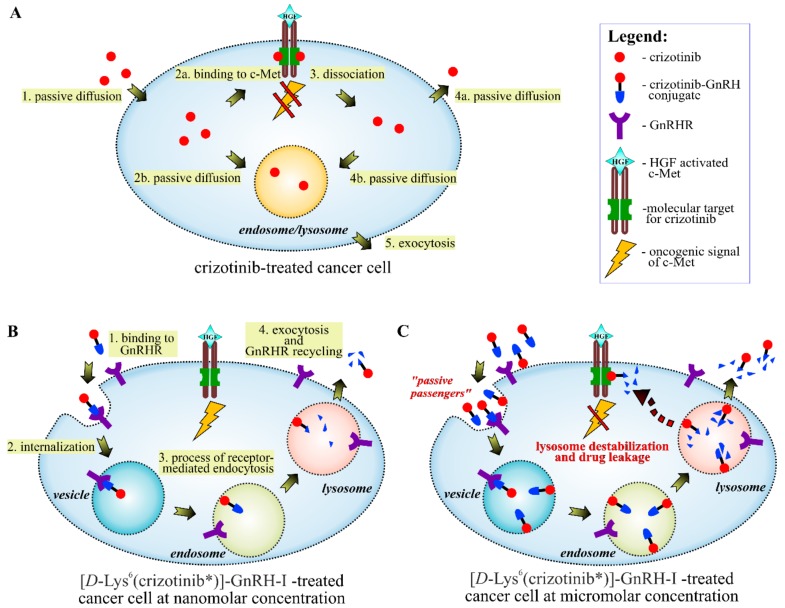
Intracellular trafficking of crizotinib* and [d-Lys^6^(crizotinib*)]–GnRH-I. (**A**) Crizotinib is able to reach the ATP-binding site of c-Met by passive diffusion and to effectively inhibit the c-Met-mediated oncogenic signaling, while **only** a proportion of the drug accumulates in the lysosomes. (**B**) The GnRHR-mediated endocytosis leads to the lysosomal sequestration of GnRHR-targeted crizotinib* at the nanomolar concentration range; meanwhile, crizotinib* bypasses c-Met. (**C**) One possible explanation for the c-Met inhibition and the cell viability inhibition effect of [d-Lys^6^(crizotinib*)]–GnRH-I at the micromolar concentration range is that during the GnRHR internalization, non–GnRHR-bound conjugates might loaded into the vesicles as “passive passengers”. High lysosomal accumulation of these passive passengers might destabilize the membrane of the lysosomes, leading to drug leakage and cell death.

The majority of drugs, which have been developed for oral use, have a weak basic character in order to achieve suitable pharmacokinetic properties. Unfortunately, these drugs may accumulate in the lysosomes with passive diffusion, a process which is enhanced further by transporter proteins [34]. While untargeted drugs (e.g., free crizotinib) have the chance to reach their site-specific molecular targets before they are trapped in the lysosomes (Figure 9A), GnRH-delivered drugs are transported directly into the endosomes/lysosomes via GnRHR-mediated endocytosis (Figure 9B). In the endosome, GnRH conjugates dissociate from GnRHR due to the decreasing pH. Subsequently, the GnRH peptide undergoes enzymatic degradation in the lysosome, which creates an opportunity to release the conjugated drug in its free form. In context with the lysosomal accumulation of targeted drugs, several enzymatically degradable spacers (self-immolative spacers) have been developed to release the drug in the lysosomes [35]. However, the results of the PAMPA assay demonstrated that GnRH-conjugated crizotinib analogues could barely escape from either endosomes (pH: ~6) or lysosomes (pH: ~4.8) even in their free forms (Figure 7). This result highlights that reduced permeability in the endosomes/lysosomes could be a general problem for targeted lysosomotropic drugs, which cannot be solved by drug-releasing spacers.

We previously reported that cellular GnRH uptake does not depend solely on the GnRHR-mediated transport at micromolar concentrations or above [18]. Quantitative analysis of [d-Lys^6^(FITC)]–GnRH-I in EBC-1 cells also demonstrated that the amount of the conjugate in the treated cells increased in proportion to the applied concentration between 0.1 and 10 µM. However, previous radioligand-binding and dose–response studies on MCF-7 human breast cancer cells [27], αT3-1 human gonadotrope cells [36], and human GnRHR-transfected COS-7 cells [37,38] have suggested that the applied concentration range of [d-Lys^6^(FITC)]–GnRH-I (10^−7^–10^−5^ M) on EBC-1 cells was several orders of magnitude higher than the ypothetical GnRHR saturation concentration (<10^−8^ M). Furthermore, the insatiable cellular uptake of [d-Lys^6^(FITC)]–GnRH-I was obvious even after 2 h of treatment, which strongly suggested the presence of a GnRHR-independent uptake mechanism in EBC-1 cells (Figure 3C). 

One possible explanation of the enhanced GnRH uptake could be that during the internalization of GnRHR and other endocytic events (e.g., micropinocytosis [39]), non-receptor-bound conjugates are also able to get into the vesicles with the cell culture medium and/or with the plasma membrane as “passive passengers” (Figure 9C). These passive passengers might become predominant in the treated cells at higher concentrations. 

There is evidence that lysosomotropic/amphiphilic drugs are able to destabilize the membranes of lysosomes, leading to drug escape and even to apoptosis at higher doses [40,41,42]. In addition, Zhitomirsky et al. also reported that lysosomal accumulation of anticancer drugs induces lysosomal membrane permeabilization (LMP) and consequent activation of transcription factor EB (TFEB) [43]. Since TFEB is a master regulator of lysosomal biogenesis and promotes lysosomal exocytosis, it could be that lysosomal exocytosis is the major intracellular mechanism of clearance for GnRH-delivered crizotinib. 

To strengthen our hypothesis on the lysosomal accumulation of [d-Lys^6^(crizotinib*)]–GnRH-I and the consequent LMP in EBC-1 cells, a galectin puntca assay was applied. Intracellular puncta formation of galectin-1 and galectin-3 has been reported to be a sensitive and robust marker of LMP [44]. Our galectin puntca assay provided evidence that both crizotinib* and [d-Lys^6^(crizotinib*)]–GnRH-I accumulated in the lysosomes and caused LMP in a concentration-dependent manner, as it shown in Figure 8. 

Based on the above-mentioned results, we concluded that EBC-1 cells are able to get rid of [d-Lys^6^(crizotinib*)]–GnRH-I at nanomolar concentrations by lysosomal exocytosis, and thus could survive. However, at micromolar doses, highly accumulated [d-Lys^6^(crizotinib*)]–GnRH-I causes LMP and drug leakage (Figure 8), which could explain the c-Met inhibitory effect and the consequent viability reduction of EBC-1 cells (Figure 4A and Figure 5). The slightly decreased efficacy of the [d-Lys^6^(MJ55*)]–GnRH-I conjugate compared to free MJ55* can also be partially explained by lysosomal sequestration: despite the fast release of MJ55* from the conjugate, the effect of the GnRHR-associated conjugates is abrogated (Figure 4A).

In general, we hypothesize that the efficacy of other lysosomotropic/amphiphilic drug-bearing GnRH conjugates (e.g., daunorubicin–GnRH [45,46]) is also mediated by LMP at micromolar concentrations, but this presumption requires further investigation. On the other hand, we also note that to achieve and maintain micromolar concentrations of GnRH conjugates in the tumor microenvironment could be more than challenging in vivo. Interestingly, the importance of lysosomal trapping and endosomal/lysosomal drug escape has not been previously highlighted in the context of GnRHR targeting. 

In summary, we demonstrated that *MET*-amplified EBC-1 NSCLC cells highly express GnRHR, and are very sensitive for crizotinib*. Experiments with fluorescently labeled GnRH-I in EBC-1 cells corroborated that GnRH conjugates are delivered into the lysosomes via GnRHR-mediated endocytosis. Our novel crizotinib analogue MJ55* had similar c-Met inhibition potential to crizotinib*; furthermore, the introduced hydroxyl group represents new possibilities for drug targeting. The [d-Lys^6^(MJ55*)]–GnRH-I conjugate resulted in a potent effect on EBC-1 NSCLC cells, but also revealed the drawback of the ester bond, as the early-released MJ55* reduced the selectivity of the GnRHR targeting. On the other hand, in vitro pharmacological profiling of the stable amide-bond-containing [d-Lys^6^(crizotinib*)]–GnRH-I revealed that the membrane of the lysosome could be a significant intracellular barrier for GnRHR-targeted drugs. In addition, the limited amount of GnRHR on the plasma membranes of cancer cells might be another restrictive factor, which is a less investigated part of GnRHR-targeted drug delivery and should be further investigated. Altogether, these findings highlight that the individual pharmacokinetics of targeted molecules necessitate more specific drug development than classical drug design, and there are still numerous unanswered questions.

## 4. Materials and Methods

### 4.1. Synthesis of Compounds

The syntheses of [d-Lys^6^]–GnRH-I peptide, and the synthetic routes for crizotinib* and MJ55* drugs, and [d-Lys^6^(crizotinib*)]–GnRH-I and [d-Lys^6^(MJ55*)]–GnRH-I drug–peptide conjugates are detailed in the Supplementary Information, Section S1. The relevant analytical methods and structural determinations can be found in the Supplementary Information, Section S2 (HPLC purity: Figure S1, HRMS: Table S1, ^1^H- and ^13^C-NMR: Figure S4). 

### 4.2. Cell Cultures

EBC-1 cell line was obtained from the Japanese Collection of Research Bioresources Cell Bank (JCRB, Solana Beach, CA 92075 USA) and cultured in Eagle’s minimum essential medium (EMEM) (Lonza, Basel, Switzerland, cat. no.: BE12-611F,) supplemented with 1% MycoZap (Lonza, cat. no.: VZA-2012) and 10% fetal bovine serum (FBS) (Thermo Fisher Scientific, Waltham, MA, USA, cat. no.: 10500-064).

LNCaP cell line was purchased from American Type Culture Collection (Manassas, VA, USA), and maintained in Roswell Park Memorial Institute (RPMI-1640) Medium (Thermo Fisher Scientific, cat. no.: 11875-093) supplemented with 1% MycoZap, 2 mM glutamine (Lonza, cat. no.: BE17-605E) and 10% FBS.

Primary human skin fibroblast cells (fibroblast cells) were gifted by the Institute of Genomic Medicine and Rare Disorders, Semmelweis University (Budapest, Hungary). Fibroblast cells were cultured in Dulbecco’s modified Eagle’s medium (DMEM) (Thermo Fisher Scientific, cat. no.: 41965039) supplemented with 10% FBS (Thermo Fisher Scientific), 1% penicillin–streptomycin (Thermo Fisher Scientific, cat. no.: 15070063) and 1% MEM Non-Essential Amino Acids Solution (Thermo Fisher Scientific, cat. no.: 11140035). Cell cultures were grown in vitro at 37 °C, 95% air and 5% CO_2_ atmosphere.

### 4.3. Western Blot

To analyze the effect of the compounds on c-Met phosphorylation, EBC-1 cells were seeded in six well plates and grown to 90% confluence. Cells were then treated with 0.1 µM [d-Lys^6^]–GnRH-I, crizotinib*, MJ55*, and [d-Lys^6^(MJ55*)]–GnRH-I in complemented EMEM. In the case of [d-Lys^6^(crizotinib*)]–GnRH-I-treated cells, 0.1 µM, 1 µM, and 10 µM concentrations were applied. Cells were incubated with the compounds for 6 h at 37 °C in a CO_2_ incubator. After the incubation, western blot analysis was performed. To investigate GnRH-R protein level, cells were seeded in six well plates and grown to 90% confluence, then western blot analysis was applied.

Western blot analysis was performed as follows: cells were washed with ice-cold PBS (Lonza, cat. no.: 17-516F) and lysed with radioimmunoprecipitation assay (RIPA) buffer (50 mM Tris (pH 7.4), 150 mM NaCl, 1% (*v*/*v*) NP-40, 0.5% (*m*/*v*) sodium deoxycholate, 0.1% (*m*/*v*) sodium dodecyl sulphate, 2 mM EDTA, and 2 mM EGTA (reagents were obtained from Merck Millipore, Burlington, MA, USA) complemented with phosphatase and protease inhibitor cocktail (Merck Millipore, cat. no.: 524629 and 539134). Lysates were sonicated three times for 10 s and incubated on ice for 30 min. After centrifugation (13,000× *g*, 4 °C, 15 min), protein concentration was determined by Bradford assay. Equal amounts of protein (20 μg) were subjected to SDS-PAGE and electrotransferred to polyvinylidene difluoride membranes (Bio-Rad, cat. no.: 162177, Budapest, Hungary). The membranes were probed with primary antibodies GnRH-I R (Proteintech, Rosemont, IL, USA, cat. no.: 19950-1-AP, dilution: 1:4000), pTyr1234/1235-Met (Cell Signaling Technology, Leiden, The Netherlands, cat.no.: 3077, dilution: 1:6000), Met (Cell Signaling Technology, cat. no.: 3148, dilution: 1:8000), pThr202/Tyr204-Erk1/2 (Cell Signaling Technology, cat. no.: 4370, dilution: 1:2000), Erk1/2 (Cell Signaling Technology, cat. no.: 9107, dilution: 1:4000), and α-tubulin (Merck Millipore, cat. no.: T9026, dilution: 1:40,000)) at 4 °C overnight, and then with horse radish peroxidase conjugated secondary antibody (Cell Signaling Technology, anti-rabbit IgG—cat.no.: 7074, dilution: 1:2000, anti-mouse IgG—cat. no.: 7076, dilution: 1:10,000) for 1 h at room temperature. Bands were visualized via Enhanced Chemiluminescence detection system (Western Lightning ECL Pro, PerkinElmer, Waltham, MA, USA, cat. no.: NEL122001EA).

### 4.4. Immunofluorescence Staining of GnRHR

Cells were seeded in eight well Ibidi^®^ μ-Slide microscopic slides (2 × 10^4^ cells/well) and allowed to adhere for 48 h. Cells were fixed with 4% paraformaldehyde for 10 min and washed twice with PBS (Lonza). Cells were permeabilized with 0.1% (*v*/*v*) Triton X-100 PBS solution for 15 min to determine the total GnRHR protein level. When only the membrane GnRHR was investigated, the permeabilization step was skipped. For blocking, 5% BSA (Merck Millipore, cat. no.: A7906) in PBS was used for 30 min. Cells were incubated with hGnRH-I-R primary antibody (Proteintech™, 19950-1-AP, dilution: 1:100) for 1 h at 25 °C, followed by Alexa Fluor 594 conjugated secondary antibody (Jackson ImmunoResearch Inc., West Grove, PA, USA, cat. no.: 111-585-003, dilution: 1:300) for 1 h at 25 °C. DRAQ5 (Thermo Fisher Scientific, cat. no.: 62254) fluorescent probe solution (final concentration: 10 µM) was added to the cells for 15 min. Cells were washed three times with PBS, and a few drops of mounting medium (Merck Millipore, cat. no.: F4680-25ML) were added. Images of cells were acquired with a confocal laser microscope (Zeiss Confocal LSM 710, Carl Zeiss AG, Oberkochen, Germany). [Objective: Plan-Apochromat 63×/1.40 Oil DIC M27. Pinhole: 1.00 AU. Laser Wavelength: 543 nm and 633 nm. Detection wavelength: 593–631 nm; 676–735 nm)].

### 4.5. Intracellular Localization of [d-Lys^6^(FITC)]–GnRH-I

EBC-1 cells were seeded in eight well Ibidi^®^ μ-Slide microscopic slides (2 × 10^4^ cells/well) and allowed to adhere for 48 h. Cells were then treated with 10 µM [d-Lys^6^(FITC)]–GnRH-I in complemented EMEM (Lonza) and incubated in a humidified, 5% CO_2_ atmosphere incubator for 24 h at 37 °C. 

After [d-Lys^6^(FITC)]–GnRH-I treatment, cells were fixed using 4% paraformaldehyde for 10 min and nuclei were counterstained with DRAQ5 for 15 min. Images (in Figure 3D) were acquired by Zeiss Confocal LSM 710 microscope.

To investigate colocalization of [d-Lys^6^(FITC)]–GnRH-I with lysosomes (in Figure 6), after [d-Lys^6^(FITC)]–GnRH-I treatment (24 h, 10 µM), Lysoview 633 dye was added to the treating medium and the cells were incubated for a further 1 h. After incubation, cells were washed twice with EMEM. Images of the living cells were acquired with a Zeiss Confocal LSM 710 microscope [Objective: Plan-Apochromat 63×/1.40 Oil DIC M27. Pinhole: 1.95 AU. Laser wavelength: 488 nm and 633 nm. Detection wavelength: 496–573 nm; 633–693 nm)]. 

### 4.6. Quantitative Analysis of [d-Lys^6^(FITC)]–GnRH-I in EBC-1 Cells

2 × 10^4^ EBC-1 cells per well were seeded onto 24 well plates (VWR) and allowed to adhere for 48 h. Cells were treated with [d-Lys^6^(FITC)]–GnRH-I in complemented EMEM (Lonza) at 0.1, 1, and 10 µM, and incubated in a humidified, 5% CO_2_ atmosphere incubator for 2, 6, 24, and 72 h at 37 °C.

After incubation, supernatant was discarded and the cells were washed twice with PBS (Lonza). Trypsin (Thermo Fisher Scientific, cat. no.: 25300062) was added for 10 min at 37 °C. Cells were pelleted (250× *g*, 4 min, 4 °C) in flow cytometry tubes, resuspended in 300 µL PBS and kept at 4 °C for the rest of the experiment. The fluorescence intensity of the conjugate-stained cells was analysed using a FACS Calibur flow cytometer (BD Biosciences, San Jose, CA) using channel FL1. Data were evaluated by CellQuest Pro software (BD Biosciences). The relative median fluorescent intensity (MFI) values (based on DMSO control) were calculated. 

Calculation formula:

$$relative\;MFI\;\left(compound;\left[x\left(h\right);y\left(µM\right)\right]\right)=\frac{MFI\;\left(compound;\left[x\left(h\right);y\left(µM\right)\right]\right)}{MFI\;\left(DMSO\;control;\left[x\left(h\right)\right]\right)}$$

*x* time (h)
*y* concentration (µM)

### 4.7. Cell Viability Assay

Effects of the synthesized compounds on EBC-1 and fibroblast cell viability was measured via CellTiter-Glo^®^ luminescent cell viability assay (Promega, Madison, WI, USA) according to the manufacturer’s instructions. Briefly, cells were plated at 1000 cells/well onto a flat-bottomed, white 96 well plate (BRANDplates, cat. no.: 781965). After 24 h, cells were treated for 72 h with either the desired 4-fold serial diluted compound concentrations or vehicle (0.2% DMSO) control. After the treatment, the luminescence signal was recorded using a microplate reader (BioTek Synergy 2 Multi-Mode Reader, BioTek, Winooski, VT, USA). Dose–response curves (using a non-linear regression model) were generated and IC_50_ values were determined using Graph Pad Prism 5.02 software (GraphPad Software, San Diego, CA, USA).

### 4.8. In Vitro Inhibition of Recombinant c-Met Kinase

Active, recombinant c-Met enzyme (3 µg/mL, ProQinase, cat. no.: 0171-0000-1, Lot: 012) was incubated with ATP (3.1 µM (K_M[ATP]_), Promega, cat. no.: V6930), peptide substrate (0,25 mg/mL, poly GT, Merck Millipore, cat. no.: PO275), and compounds at the indicated concentrations. Reaction buffer was prepared according to ProQinase’s recommendation [60 mM HEPES, 3 mM MgCl_2_, 3 mM MnCl_2_, 3 µM Na_3_VO_4_, 1.2 mM DTT (dithiothreitol), and 50 µg/mL PEG_20,000,_ (reagents were obtained from Merck Millipore)]. Enzyme activity was assayed in 384 well, low-volume white microtiter plates (Corning^®^, Merck Millipore, cat. no.: CLS4513-50EA). Compounds were preincubated with c-Met kinase for 30 min at room temperature and reactions were started by adding the substrate–ATP mixture. Reaction time was 60 min at room temperature. Enzyme reaction was stopped using ADP-Glo™ Kinase Assay (Promega, cat. no.: V6930) according to the manufacturer’s instructions (40 min incubation with ADP-Glo™ Reagent, 30 min incubation with Kinase detection reagent at 25 °C). All measurements were performed using a Synergy 2 Multi-Mode Reader (BioTek, Winooski, VT, USA). Raw enzyme activity values were converted to relative activity data using positive and negative controls. Determination of IC_50_ values was performed using GraphPad Prism 5.02 software.

### 4.9. Stability Experiment

Stock solutions of compounds in DMSO were diluted to 10 µM in complemented EMEM (Lonza). Samples were incubated for 2, 6, 24, and 72 h at 37 °C. After the incubation, acetonitrile (VWR, cat. no.: 83639.320) was added to the solutions (1:1 ratio, to achieve 50% acetonitrile content). The samples were vortexed and centrifuged (13,000× *g*, 10 min). Supernatants were measured by HPLC-UV. HPLC method and chromatograms are available in the Supplementary Information. Stability was calculated using the external standard method. Decay curves were generated and half-life was calculated by GraphPad Prism 5.02 software. 

### 4.10. PAMPA

An artificial-membrane-based system (BD Gentest™ Pre-Coated PAMPA plate, Corning, cat. no.: 353015) was used to determine the pH-dependent membrane permeability of the compounds. In this method, the protocol provided by the manufacturer was followed with minor modifications. Four different pH values were applied per compound. The pH of the PBS solutions (Lonza) was adjusted to 4.6, 6.0, 7.4, and 8.0 using 1 M HCl or 1 M NaOH. Stock solutions of compounds in DMSO were diluted to 100 µM in each PBS. PBS solutions were loaded into the donor wells, but the acceptor wells were filled with physiological PBS (pH = 7.4). The system was gently shaken (BioSan Thermo-Shaker PST-60HL) for 5 h at 25 °C. Samples derived from the donor and the acceptor wells were measured using a BioTek Synergy 2 Multi-Mode Reader at 280 nm. Calculation formula and the calculated data are available in the Supplementary Information, Section S4.

### 4.11. Galectin Puncta Assay

EBC-1 cells were seeded on eight well Ibidi^®^ μ-Slide microscopic slides (2 × 10^4^ cells/well) and allowed to adhere for 48 h. Cells were treated with 10 µM crizotinib*, 0.1 µM, 1 µM, 10 µM, and 100 µM [d-Lys^6^(crizotinib*)]–GnRH-I in serum-free EMEM. Untreated (0.2% DMSO in EMEM) cells were used as a negative control. Cells were incubated with the compounds for 8 h at 37 °C in a CO_2_ incubator. Cells were fixed with 4% paraformaldehyde for 10 min and washed twice with PBS (Lonza). Cells were permeabilized with 0.1% (*v*/*v*) Triton X-100 PBS solution for 10 min. After blocking (5% *v*/*v* BSA in PBS), cells were incubated with anti-galectin 3 (Merck, cat. no.: SAB4501746, dilution: 1:100) and anti-galectin-1 (Santa Cruz Biotechnology, cat. no.: sc-166618, dilution: 1:100) primary antibodies overnight at 25 °C, followed by Alexa-Fluor-488-conjugated (Jackson ImmunoResearch., cat. no.: 111-545-003, dilution: 1:500) and Alexa-Fluor-594-conjugated (Jackson ImmunoResearch, cat. no.: 115-585-003, dilution: 1:250) secondary antibodies for 1 h at 25 °C. DRAQ5 (10 µM) was added to the cells for 15 min. Cells were washed three times with PBS and a few drops of mounting media (Merck Millipore) were added. Images of cells were acquired with a confocal laser microscope (Zeiss Confocal LSM 710). [Objective: Plan-Apochromat 63x/1.40 Oil DIC M27. Pinhole: 1.01 AU. Laser wavelength: 488 nm, 543 nm and 633 nm. Detection wavelength: 504–539 nm; 602–651 nm; 694–758 nm)].

### 4.12. Radioligand-Binding Assay

The receptor binding affinities of crizotinib–GnRH conjugates were determined by displacement analyses using radioiodinated GnRH-I agonist triptorelin ([^125^I]-[d-Trp^6^]–GnRH-I) on human pituitary and human prostate cancer cells. The collection and use of the tissue samples for our studies were approved by the local Institutional Ethics Committee. Normal human pituitary (anterior lobe) tissue was collected at autopsy and the human prostate cancer specimen was obtained from a patient at the time of initial surgical treatment. The ligand competition assay was performed as described previously [19].

## 5. Conclusions

Based on the literature data, we designed and synthesized novel crizotinib analogues and successfully conjugated them with [d-Lys^6^]–GnRH-I peptide. Although these crizotinib–GnRH conjugates had potent inhibitory potential on recombinant c-Met kinase and they were able to bind to GnRH-R, the benefit of GnRHR targeting was not clearly proven on the *MET*-amplified and GnRHR-overexpressing EBC-1 NSCLC cells in vitro. We concluded that the decreased efficacy of crizotinib–GnRH conjugates could be explained by the mechanism of receptor-mediated endocytosis. Intracellular trafficking of GnRH/GnRHR directs conjugated crizotinib into the lysosomes, and hence crizotinib bypasses the ATP-binding site of c-Met. At nanomolar doses, GnRH-delivered crizotinib is trapped in the lysosome due to acidic pH and triggers lysosomal exocytosis. At micromolar doses, the observed c-Met inhibition and cell viability reduction effects of [d-Lys^6^(crizotinib*)]–GnRH-I could be explained by crizotinib-induced LMP.

In general, we found that lysosomal sequestration of GnRH-delivered drugs partially explained the limited success of anti-cancer GnRH conjugates retrospectively. In order to design more effective GnRH-based drug delivery systems, the importance of drug escape from the lysosomes should be taken into account. Therefore, we suppose that drugs with enhanced membrane permeability at mildly acidic pH could be more appropriate for GnRHR-targeted drug delivery. In the case of compounds with poor permeability, incorporation of a membrane-penetrative spacer [47] might be an effective solution to enhance the drug’s escape from the lysosomes. Nevertheless, it has been reported that targeting the lysosomes could be a promising strategy for cancer therapy [48,49]. Thereby, the lysosome-directed transport of GnRHR might even be advantageous, for example in the case of GnRH-conjugated lysosome inhibitors, and also for drugs with no site-specific molecular target (e.g., protoporphyrin derivatives [50,51]).

## Figures and Tables

**Table 1:** Inhibition of [^125^I]-[d-Trp^6^]–GnRH-I binding to the membranes of human pituitary and human prostate cancer cells by GnRH derivatives.

Compound	IC_50_ Values (nM) ^1^	
	Human Pituitary	Human Prostate Cancer
[d-Lys^6^]GnRH-I (vehicle)	6.44 ± 1.0	4.31 ± 0.8
[d-Lys^6^(FITC)]–GnRH-I	27.2 ± 2.8	18.3 ± 2.9
[d-Lys^6^(crizotinib*)]–GnRH-I	34.6 ± 4.2	39.5 ± 1.7
[d-Lys^6^(MJ55*)]–GnRH-I	21.7 ± 1.4	29.7 ± 3.1

^1^ IC_50_ values were calculated via computerized curve-fitting program from displacement experiments, as described previously [19]. IC_50_ is defined as the dose causing 50% inhibition of specific binding of [^125^I]-[d-Trp^6^]–GnRH-I to the membranes. Values are mean ± standard deviation (SD) of two to three independent experiments.

## Supplementary Materials

Supplementary materials can be found at https://www.mdpi.com/1422-0067/20/22/5590/s1.

## Abbreviations

ALK	anaplastic lymphoma kinase
BOC	tert-butyloxycarbonyl-protecting group
CLSM	confocal laser scanning microscopy
c-Met	hepatocyte growth factor receptor
COMU	(1-Cyano-2-ethoxy-2-oxoethylidenaminooxy)dimethylamino-morpholino-carbenium hexafluorophosphate
crizotinib	(R)-crizotinib, (Xalkori^®^, Pfizer)
crizotinib*	racemic mixture of (R)-crizotinib and (S)-crizotinib
EMEM	Eagle’s minimum essential medium
FBS	Fetal bovine serum
fibroblast cells	human primary human skin fibroblast cells
galectin-1	lectin galactoside binding soluble 1
galectin-3	lectin galactoside binding soluble 3
HRMS	high resolution mass spectrometry
LMP	lysosomal membrane permeabilization
MJ55*	racemic mixture of (R)-MJ55 and (S)-MJ55
NSCLC	non-small cell lung cancer
Pd(dppf)Cl_2_	[1,1′-Bis(diphenylphosphino)ferrocene]dichloropalladium(II)
ROS-1	ROS proto-oncogene 1, receptor tyrosine kinase
RTK	receptor tyrosine kinase
SD	standard deviation
TEA	triethylamine
TFEB	transcription factor EB
THF	tetrahydrofuran
