# Supplementary material for: Novel Crizotinib–GnRH Conjugates Revealed the Significance of Lysosomal Trapping in GnRH-Based Drug Delivery Systems
Source: Int J Mol Sci. 2019 Nov 8;20(22):5590. doi: 10.3390/ijms20225590 (PMC6888004; doi:10.3390/ijms20225590)
Supplement: Supplementary file 1 [file ijms-20-05590-s001.pdf]

Supplementary file

S1. Synthesis of compounds

All amino acid derivatives and reagents were purchased from Millipore Sigma (Burlington, MA, USA). The solvents were purchased from Reanal Ltd. (Budapest, Hungary). All reagents and solvents were reagent grade or higher purity. Analytical methods are detailed in the Supplementary file S1. (HPLC purity: Figure A1, HRMS: Table A1, ^{1}H- and ^{13}C-NMR: Figure A4)

Compound 1. {6-[bis(tert-butoxycarbonyl)amino]-5-[1-(2,6-dichloro-3-fluorophenyl)ethoxy]pyridin-3-yl}boronic acid

The synthesis of 1 was carried out by following the literature data 11.

Compound 2. 4-(4-iodo-1H-pyrazol-1-yl)piperidine

The synthesis of 2 was carried out by following the literature data 11.

Compound 3. di-tert-butyl {3-[1-(2,6-dichloro-3-fluorophenyl)ethoxy]-5-(1-piperidin-4-yl-1H-pyrazol-4-yl)pyridin-2-yl}imidodicarbonate

To the mixture of compound **1** (500 mg, 0.797 mmol) and compound **2** (265 mg 0.956 mmol) in DMSO (10 mL) was added 2M Cs_2CO_3 aqueous solution (1.2 mL). The mixture was purged with nitrogen, 5 m/m% Pd(dppf)Cl_2 (25 mg) was added. The resulting mixture was stirred at 70°C for 3 hours under nitrogen atmosphere, then cooled to 5°C. To this cooled mixture 10% (m/V) Na_2CO_3 solution (25 mL) was added, stirred for 15 minutes and filtered. The precipitate was dissolved in diethyl ether (25 mL) and the solution was filtered, and sequentially washed with 10% (m/V) Na_2CO_3 solution (3 x 25 mL) and brine (25 mL). The organic phase was concentrated under vacuo. The crude product was purified by silica gel column, eluting with the mixture of $CHCl_3$ (eluent A) and ammonia solution 2.0 M in methanol (eluent B) (gradient of eluent B 5% to 20%). The combined fractions were concentrated under vacuum to give compound **3** as white solid foam: (250 mg, 48%). HPLC (method A) t_R=5.63 min. LCMS m/z: 650.23 $(M + H)^+$. ^{1}H NMR: 8.29 (s, 1H, H-15); 8.24 (d, *J*=1.7 Hz, 1H, H-11); 7.87 (s, 1H, H-16); 7.61 (d, *J*=1.7 Hz, 1H, H-11); 7.51 (dd, *J*=8.8 Hz and 4.9 Hz, 1H, H-4); 7.41 (t, *J*=8.8 Hz, 1H, H-3); 6.24 (qa, *J*=6.7 Hz, 1H, H-7); 4.18 (tt, *J*=11.4 Hz and 4.2 Hz, 1H, H-17); 3.03 (br ~d, *J*~12 Hz, 2H, H-$19_{equatorial}$); 2.57 (br ~t, *J*~12 Hz, 2H, H-19_{axial}); 1.95 (br ~d, *J*~12 Hz, 2H, H-$18_{equatorial}$); 1.77 (qad, *J*=11.9 Hz and 4.2 Hz, 2H, H-18_{axial}); 1.73 (d, *J*=6.7 Hz, 3H, H-8); 1.33 and 1.15 [2xbr s, 2x9H, 2x$(C\underline{H}_3)_3$C-CO-O-]. ^{13}C NMR: 157.3 (d, *J*=247.7 Hz, C-2); 150.7 and 150.6 [2x $(CH_3)_3$C-$\underline{C}$O-O-]; 148.6 (C-10); 140.5 (C-9); 137.2 (C-6); 137.0 (C-11); 136.4 (C-16); 130.9 (d, *J*=5.2 Hz, C-4); 129.4 (d, *J*=3.2 Hz, C-5); 127.1 (C-15); 121.7 (d, *J*=19.3 Hz, C-1); 118.1 (two coalesced lines, C-12 and C-14); 117.9 (d, *J*=23.4 Hz, C-3); 115.0 (C-13); 82.2 and 81.9 [2x $(CH_3)_3\underline{C}$-CO-O-]; 73.3 (C-7); 59.7 (C-17); 45.3 (C-19); 33.7 (C-18); 18.7 (C-8).

Compound 4. 3-[1-(2,6-dichloro-3-fluorophenyl)ethoxy]-5-(1-piperidin-4-yl-1H-pyrazol-4-yl)pyridin-2-amine (racemic crizotinib)

To the compound **3** (130 mg, 0.200 mmol) 5 mL TFA/water mixture (9:1 ratio) was added at 4°C and the solution was stirred at room temperature for 2 h. The solvent was concentrated under vacuo. The crude product was dissolved in 1:3 mixture of acetonitrile/water and purified by semi-preparative HPLC, using Phenomenex Gemini C18 column (150 mm/21.4 mm, 5 μm, 110Å). Eluent A: water containing 0.1% TFA. Eluent B: 9:1 mixture of acetonitrile/water containing 0,1% TFA. Gradient: 0 min: 0% B, 5 min: 25% B, 50 min: 40% B. The combined fractions were concentrated under vacuo, and the product was dissolved in THF EtOAc 1:1 mixture (5 mL), then 10% m/V NaOH solution (5mL) was added, the phases were separated. The aqueous phase was extracted with EtOAc (2x3 mL). The combined organic phase was washed with brine, dried over Na_2SO_4, filtered and concentrated to give compound **4** (racemic crizotinib) as white powder. (70 mg, 78%). HPLC (method A) t_R=4.38 min. LCMS m/z: 450.13 $(M + H)^+$. ^{1}H NMR: 7.88

(s, 1H, H-15); 7.72 (d, *J*=1.7 Hz, 1H, H-11); 7.52 (overlapping s and dd, *J*=8.7 Hz and 4.8 Hz, 2H, H-16 and H-4, resp.); 7.39 (t, *J*=8.7 Hz, 1H, H-3); 6.85 (d, *J*=1.7 Hz, 1H, H-13); 6.04 (qa, *J*=6.7 Hz, 1H, H-7); 5.63 (s, 2H, $N\underline{H}_2$); 4.32 (tt, *J*=11.2 Hz and 4.1 Hz, 1H, H-17); 3.24 and 2.88 (2xm, 2x2H, H-19); 2.06 and 1.94 (2xm, 2x2H, H-18); 1.75 (d, *J*=6.7 Hz, 3H, H-8). ^{13}C NMR: 157.3 (d, *J*=247.7 Hz, C-2); 150.0 (C-10); 139.3 (C-9); 137.3 (C-6); 136.0 (C-11); 135.4 (C-16); 131.0 (d, *J*=5.1 Hz, C-4); 129.2 (d, *J*=3.3 Hz, C-5); 124.3 (C-15); 121.5 (d, *J*=19.3 Hz, C-1); 119.8 (C-12); 117.9 (d, *J*=23.4 Hz, C-3); 117.6 (C-14); 114.9 (C-13); 72.4 (C-7); 56.8 (C-17); 43.5 (C-19); 30.7 (C-18); 19.1 (C-8).

Compound 5. 5-[4-(4-{6-[bis(tert-butoxycarbonyl)amino]-5-[1-(2,6-dichloro-3-fluorophenyl)ethoxy]pyridin-3-yl}-1H-pyrazol-1-yl)piperidin-1-yl]-5-oxopentanoic acid

Solution of compound **3** (120 mg, 0.185 mmol) in CH_2Cl_2 (6 mL) was cooled to 0°C and TEA (103 μL, 0.738 mmol) and glutaric anhydride (25 mg, 0.221 mmol) were added. The mixture was stirred at room temperature for 2 hours and the solvent was removed under vacuo. The crude product was purified by preparative RP-HPLC, the combined fractions were lyophilised to obtain compound **5** as white solid foam: (120 mg, 85%). HPLC (method A) t_R=6.94 min. LCMS m/z: 764.26 $(M + H)^+$. ^{1}H NMR: 8.34 (s, 1H, H-15); 8.24 (d, *J*=1.8 Hz, 1H, H-11); 7.90 (s, 1H, H-16); 7.63 (d, *J*=1.8 Hz, 1H, H-13); 7.51 (dd, *J*=8.8 Hz and 4.9 Hz, 1H, H-4); 7.41 (t, *J*=8.8 Hz, 1H, H-3); 6.24 (qa, *J*=6.7 Hz, 1H, H-7); 4.49 and 4.42 (partly overlapping br ~d and tt, *J*~11 Hz and *J*=11.2 Hz and 4.1 Hz, 2x1H, $H\text{-}19_{equatorial}$ and H-17, resp.); 3.94 (br ~d, *J*~11 Hz, 1H, $H\text{-}19'_{equatorial}$); 3.15 (br ~t, *J*~11 Hz, 1H, $H\text{-}19'_{axial}$); 2.71 (br~t, *J*~11 Hz, 1H, $H\text{-}19_{axial}$); 2.35 (t, *J*=6.4 Hz, 2H, H-21); 2.23 (t, *J*=6.4 Hz, 2H, H-23); 2.07 (br ~d, *J*~11 Hz, 1H, $H\text{-}18_{equatorial}$); 2.02 (br ~d, *J*~11 Hz, 1H, $H\text{-}18'_{equatorial}$); 1.82 (m, 1H, $H\text{-}18_{axial}$); 1.72 (m, 1H, $H\text{-}18'_{axial}$); 1.73 (d, *J*=6.7 Hz, 3H, H-7); 1.69 (qi, *J*=6.4 Hz, 2H, H-22); 1.33 and 1.15 [2x br s, 2x9H, 2x$(C\underline{H}_3)_3C$-CO-O-]. ^{13}C NMR: 175.2 (C-24); 170.9 (C-20); 157.5 (d, *J*=247.7 Hz, C-2); 150.8 and 150.6 [2x $(CH_3)_3C$-$\underline{C}$O-O-]; 148.7 (C-9); 140.6 (C-10); 137.2 (two coalesced lines, C-6 and C-11); 136.8 (C-16); 131.0 (d, *J*=5.1 Hz, C-4); 130.1 (C-12); 129.5 (d, *J*=3.3 Hz, C-5); 126.6 (C-15); 121.8 (d, *J*=19.3 Hz, C-1); 118.3 (C-13); 118.2 (C-14); 118.1 (d, *J*=23.4 Hz, C-3); 82.3 and 82.0 [2x $(CH_3)_3\underline{C}$-CO-O-]; 73.5 (C-7); 59.0 (C-17); 44.2 (C-19′); 40.5 (C-19); 33.6 (C-23); 33.3 (C-18); 32.4 (C-18′); 32.1 (C-21); 28.1 and 27.8 [2x $(\underline{C}H_3)_3C$-CO-O-]; 20.9 (C-22); 18.7 (C-8).

Compound 6. 2-[4-(4-iodo-1H-pyrazol-1-yl)piperidin-1-yl]ethanol

To the solution of compound **2** (370 mg, 1.335 mmol) in dry THF (10 mL) K_2CO_3 (921 mg, 6.676 mmol) was added and the mixture was stirred for 30 minutes at reflux temperature under nitrogen atmosphere. Bromoethanol (469 μL, 2.670 mmol) was added, and the reaction mixture was stirred for 24 h, at reflux temperature, under nitrogen atmosphere. The reaction mixture was cooled to ambient temperature, filtered, and the precipitate was washed with THF. The filtrate was concentrated under vacuum. The crude product was dissolved in CH_2Cl_2 (10 mL), then cooled to 0°C and 4 M HCl-dioxane (1 mL) was added. The white suspension was filtered off, and the crude product was dissolved in H_2O (10 mL) and washed with diethyl ether (5 mL).

The pH of the aqueous layer was adjusted to >10 by adding 10% (m/V) NaOH solution, and then the aqueous portion was extracted with CH_2Cl_2 (2*5 mL). The organic layer was dried over Na_2SO_4, filtered and concentrated by vacuum: (370 mg, 86%). HPLC (method A) t_R=4.09 min. LCMS m/z: 322.04 $(M + H)^+$. ^{1}H NMR: 7.92 (s, 1H, H-15); 7.46 (s, 1H, H-16); 4.36 (t, *J*=5.5 Hz, 1H, $O\underline{H}$); 4.08 (tt, *J*=10.8 Hz and 4.9 Hz, 1H, H-17); 3.45 (qa, *J*=5.5 Hz, 2H, H-21); 2.36 (t, *J*=5.5 Hz, 2H, H-20); 2.89 (dt, *J*=11.9 Hz and 3.2 Hz, 2H, $H\text{-}19_{equatorial}$); 2.05 (td, *J*=11.9 Hz and 3.2 Hz, 2H, $H\text{-}19_{axial}$); 1.90-1.82 (m, 4H, H-18). ^{13}C NMR: 143.4 (C-16); 132.7 (C-15); 60.5 (C-20); 59.5 (C-17); 59.2 (C-21); 56.9 (C-14); 52.9 (C-19); 32.4 (C-18).

Compound 7. di-tert-butyl (3-[1-(2,6-dichloro-3-fluorophenyl)ethoxy]-5-{1-[1-(2-hydroxyethyl)piperidin-4-yl]-1H-pyrazol-4-yl}pyridin-2-yl)imidodicarbonate

To the mixture of compound **1** (500 mg, 0.797 mmol) and compound **6** (307 mg 0.956 mmol) in DMSO (10 mL) 2M Cs_2CO_3 aqueous solution (1.2 mL) was added. In the following, the synthesis of compound **7** was performed by using similar procedure as described above (compound **3**). The obtained compound **7** is white solid foam: (290 mg, 52%). HPLC (method A) t_R=5.66 min. LCMS m/z: 694.26 $(M + H)^+$. ^{1}H NMR:

8.32 (s, 1H, H-15); 8.24 (d, *J*=1.8 Hz, 1H, H-11); 7.87 (s, 1H, H-16); 7.61 (d, *J*=1.8 Hz, 1H, H-13); 7.51 (dd, *J*=9.0 Hz and 4.9 Hz, 1H, H-4); 7.41 (t, *J*=9.0 Hz, 1H, H-3); 6.24 (qa, *J*=6.7 Hz, 1H, H-7); 4.42 (tt, *J*=11.1 Hz and 4.3 Hz, 1H, H-17); 4.36 (br ~t, *J*~5 Hz, 1H, O<u>H</u>); 3.48 (t, *J*=5.3 Hz, 2H, H-21); 2.94 (br ~d, *J*~11 Hz, 2H, H-$19_{equatorial}$); 2.39 (t, *J*=5.3 Hz, 2H, H-20); 2.11 (br ~t, *J*~11 Hz, 2H, H-19_{axial}); 2.00-1.87 (m, 2H, H-18); 1.73 (d, *J*=6.7 Hz, 3H, H-7); 1.33 and 1.15 [2x br s, 2x9H, 2x (C<u>H</u>$_3$)$_3$C-CO-O-]. ^{13}C NMR: 157.5 (d, *J*=247.7 Hz, C-2); 150.7 and 150.6 [2x (CH$_3$)$_3$C-<u>C</u>O-O-]; 148.8 (C-9); 140.7 (C-10); 137.2 (C-6); 136.8 (C-11); 136.6 (C-16); 131.0 (d, *J*=5.1 Hz, C-4); 130.1 (C-12); 129.5 (d, *J*=3.3 Hz, C-5); 126.6 (C-15); 121.9 (d, *J*=19.8 Hz, C-1); 118.2 (two coalesced lines, C-13 and C-14); 118.0 (d, *J*=23.4 Hz, C-3); 82.3 and 82.0 [2x (CH$_3$)$_3$<u>C</u>-CO-O-]; 73.5 (C-7); 60.4 (C-20); 59.6 (C-21); 59.4 (C-17); 53.1 (C-19); 32.8 (C-18); 28.3 and 28.2 [2x (<u>C</u>H$_3$)$_3$C-CO-O-]; 18.9 (C-8).

Compound 8. **2-[4-(4-{6-amino-5-[1-(2,6-dichloro-3-fluorophenyl)ethoxy]pyridin-3-yl}-1H-pyrazol-1-yl)piperidin-1-yl]ethanol**

To the compound **7** (120 mg, 0.173 mmol) 5 mL TFA/water mixture (9:1 ratio) was added at 4°C and the solution was stirred at room temperature for 2 h. In the following, the synthesis of compound **8** was performed by using similar procedure described for the synthesis of compound **4**. The obtained compound **8** (racemic MJ55) was white solid foam: (60 mg, 70%). HPLC (method A) t_R=4.37 min. LCMS m/z:.494.15 (M + H)$^+$. ^{1}H NMR: 7.89 (s, 1H, H-15); 7.71 (d, *J*=1.7 Hz, 1H, H-11); 7.51 (dd, *J*=8.8 Hz and 4.8 Hz, 1H, H-4); 7.45 (S, 1H, H-16); 7.38 (t, *J*=8.8 Hz, 1H, H-3); 6.85 (d, *J*=1.7 Hz, 1H, H-13); 6.03 (qa, *J*=6.7 Hz, 1H, H-7); 5.59 (s, 2H, N<u>H</u>$_2$); 4.38 (br s, 1H, O<u>H</u>); 4.04 (tt, *J*=11.2 Hz and 4.3 Hz, 1H, H-17); 3.48 (t, *J*=6.3 Hz, 2H, H-20); 2.92 (br ~d, *J*~12 Hz, 2H, H-$19_{equatorial}$); 2.40 (t, *J*=6.3 Hz, 2H, H-21); 2.11 (br ~t, *J*~12 Hz, 2H, H-19_{axial}); 1.99-1.82 (m, 4H, H-18); 1.75 (d, *J*=6.7 Hz, 3H, H-7). ^{13}C NMR: 157.3 (d, *J*=247.8 Hz, C-2); 149.9 (C-10); 139.3 (C-9); 137.3 (C-6); 136.0 (C-11); 135.0 (C-16); 131.0 (br ~s, C-4); 129.2 (d, *J*=3.5 Hz, C-5); 123.9 (C-15); 121.5 (d, *J*=19.4 Hz, C-1); 119.6 (C-12); 117.9 (three lines: the second one is a s, C-14; the terminal lines constitute a d, *J*=23.2 Hz, C-3); 114.9 (C-13); 72.6 (C-7); 60.5 (C-21); 59.2 (C-20); 59.0 (C-17); 52.9 (C-19); 32.5 (C-18); 19.1 (C-8).

Compound 9. **5-{2-[4-(4-{6-[bis(tert-butoxycarbonyl)amino]-5-[1-(2,6-dichloro-3-fluorophenyl)ethoxy]pyridin-3-yl}-1H-pyrazol-1-yl)piperidin-1-yl]ethoxy}-5-oxopentanoic acid**

Solution of compound **7** (120 mg, 0.173 mmol) in CH_2Cl_2 (6 mL) was cooled to 0°C and TEA (96 µL, 0.691 mmol) and glutaric anhydride (24 mg, 0.207 mmol) were added. The mixture was stirred at room temperature for 2 hours and the solvent was removed under vacuo. The crude product was purified by preparative RP-HPLC, the combined fractions were lyophilised to obtain compound **9** as white powder: (70 mg, 50%). HPLC (method A) t_R=5.79 min. LCMS m/z: 808.29 (M + H)$^+$. ^{1}H NMR: 8.33 (s, 1H, H-15); 8.27 (d, *J*=1.8 Hz, 1H, H-11); 7.88 (s, 1H, H-16); 7.65 (d, *J*=1.8 Hz, 1H, H-13); 7.51 (dd, *J*=8.9 Hz and 4.9 Hz, 1H, H-4); 7.41 (t, *J*=8.9 Hz, 1H, H-3); 6.25 (qa, *J*=6.7 Hz, 1H, H-7); 4.47 (tt, *J*=11.2 Hz and 4.5 Hz, 1H, H-17); 4.34 (br s, 2H, H-21); 3.66 (br ~d, *J*~11 Hz, 2H, H-$19_{equatorial}$); 3.42 (br s, 2H, H-20); 3.21 (br ~t, *J*~11 Hz, 2H, H-19_{axial}); 2.37 (t, *J*=6.1 Hz, 2H, H-23); 2.31-2.17 (m, 6H, H-18 and H-25); 1.74 and 1.73 (partly overlapping d, *J*=6.7 Hz and qi, *J*=6.1 Hz, 5H, H-8 and H-24); 1.34 and 1.16 [2x br s, 2x9H, 2x (C<u>H</u>$_3$)$_3$C-CO-O-]. ^{13}C NMR: 175.6 (C-26); 172.6 (C-22); 157.5 (d, *J*=247.9 Hz, C-2); 150.7 and 150.6 [2x (CH$_3$)$_3$C-<u>C</u>O-O-]; 148.7 (C-9); 140.7 (C-10); 137.2 (C-6); 136.9 (C-11); 136.5 (C-16); 131.0 (d, *J*=5.1 Hz, C-4); 130.1 (C-12); 129.5 (d, *J*=3.0 Hz, C-5); 126.5 (C-15); 122.0 (d, *J*=19.8 Hz, C-1); 118.3 (C-13); 118.2 (C-14); 118.0 (d, *J*=23.4 Hz, C-3); 82.2 and 82.0 [2x (CH$_3$)$_3$<u>C</u>-CO-O-]; 73.4 (C-7); 58.9 (C-21); 55.7 (C-17); 54.9 (C-20); 51.6 (C-19); 33.1 (C-25); 33.0 (C-23); 29.7 (C-18); 28.0 and 27.7 [2x (<u>C</u>H$_3$)$_3$C-CO-O-]; 20.2 (C-24); 18.8 (C-8).

Compound 10. **[D-Lys^6(crizotinib*)]-GnRH-I**

[D-Lys^6]-GnRH-I peptide was synthesized manually on H-Rink Amide ChemMatrix® resin using standard Fmoc/*t*Bu strategy. The following amino acid derivatives were applied: Fmoc-Gly-OH, Fmoc-Pro-OH, Fmoc-Arg(Pbf)-OH, Fmoc-Leu-OH, Fmoc-D-Lys(Alloc)-OH, Fmoc-Tyr(tBu)-OH, Fmoc-Ser(tBu)-OH, Fmoc-Trp(Boc)-OH, Fmoc-His(Trt)-OH, and pyroglutamic acid (pGlu). All couplings employed three equivalents of amino acid derivate, with COMU and NMM at room temperature, for 20 min. Coupling involved minimal preactivation times. The Fmoc group was removed with 20% piperidine

in DMF. The Alloc protecting group was removed in DCM by using tetrakis(triphenylphosphine)palladium(0) and phenylsilane under nitrogen atmosphere. After deprotection, sodium *N,N*-diethyldithiocarbamate in DMF was used for the removal of palladium.

Compound **5** (57 mg, 0.075 mmol) was dissolved in DMF then COMU (35 mg, 0.082 mmol) and NMM (16.5 µL, 0,150 mmol) were added. The mixture was added to the resin (150 mg) harbouring the selectively unprotected [D-Lys^6]-GnRH-I. The conjugation was carried out for 2 h, at 25°C. The conjugate was cleaved from the resins with TFA/phenol/water/TIPS (88:5:5:2 ratio) mixture in 2 h, at 25°C. The filtrate was precipitated in cold diethyl ether, the white precipitate was filtered and dried under vacuum to give the crude peptide conjugate. Crude conjugate was dissolved in 1:4 mixture of acetonitrile/water containing 0.1% TFA, and purified by semi-preparative HPLC using Phenomenex Gemini C18 column (150 mm/21.4 mm, 5 µm, 110 Å). Eluent A: water containing 0.1% TFA. Eluent B: 9:1 mixture of acetonitrile/water containing 0.1% TFA. Gradient: 0 min: 0% B, 5 min: 20% B, 50 min: 40% B. The combined fractions were lyophilised to obtain compound **10** ([D-Lys^6(crizotinib*)]-GnRH-I) as white solid foam (33 mg). HPLC (method A) t_R=5.45 min. LCMS m/z: 600.27 $(M + 3H)^{3+}$.

Compound 11. [D-Lys^6(MJ55*)]-GnRH-I

[D-Lys^6(MJ55*)]-GnRH-I was prepared by using similar procedure described for the synthesis of [D-Lys^6(crizotinib*)]-GnRH-I. The obtained compound **11** is white solid foam (29 mg). HPLC (method A) t_R=5.32 min. LCMS m/z: 461.46 $(M + 4H)^{4+}$.

S2. Analytical methods

S2.1 HRMS

High resolution mass spectra were acquired on a Q Exactive™ Focus, high resolution and high mass accuracy, hybrid quadrupole-orbitrap mass spectrometer (Thermo Fisher Scientific, Bremen, Germany) in the 200–2000 *m/z* range. MS data were analysed by Xcalibur™ software (Thermo Fisher Scientific). Samples were dissolved in 500 µL eluent (0.1% HCOOH in acetonitrile/water 50:50, v/v).

Table S1. High resolution mass spectrometry data of compounds

compound	formula (protonated)	$MW_{calc.}$	$MW_{meas.}$	Δppm
3	$C_{31}H_{39}Cl_2FN_5O_5$ (1+)	650.23068	650.23114	0.707
4 (crizotinib*)	$C_{21}H_{23}Cl_2FN_5O$ (1+)	450.12582	450.12678	2.132
5	$C_{36}H_{45}Cl_2FN_5O_8$ (1+)	764.26237	764.26271	0.440
6	$C_{10}H_{17}IN_3O$ (1+)	322.04108	322.04112	0.119
7	$C_{33}H_{43}Cl_2FN_5O_6$ (1+)	694.25689	694.25713	0.340
8 (MJ55*)	$C_{23}H_{27}Cl_2FN_5O_2$ (1+)	494.15204	494.15218	0.293
9	$C_{38}H_{49}Cl_2FN_5O_9$ (1+)	808.28859	808.28873	0.173
10 ([D-Lys^6(crizotinib*)]-GnRH-I)	$C_{85}H_{113}Cl_2FN_{23}O_{16}$ (3+)	600.26934	600.26937	0.050
11 ([D-Lys^6(MJ55*)]-GnRH-I)	$C_{87}H_{118}Cl_2FN_{23}O_{17}$ (4+)	461.46038	461.46112	1.603

S2.2 HPLC measurements

High performance liquid chromatograms were acquired on a Jasco HPLC system (ABL&E-JASCO Hungary Ltd., Budapest, Hungary), using Phenomenex® Gemini C18 (150/4.60 mm, 5 µm, 110 Å) column. Data were analysed by ChromPass 1.8 software. The purity was measured using Method A (Figure A1). At the stability experiment Method B was used (Figure A2 and Figure A3). Acetonitrile (cat. no.: 83639.320) and TFA (cat. no.: 153112E) were obtained for VWR.

Method A: Samples (5 mM DMSO stock solutions) were dissolved in 500 µL eluent (TFA/H_2O 1:2 mixture, containing 0.1% TFA). Injected volume: 10 µL. Linear gradient elution (0 min: 10% B, 0.2 min: 10% B, 5.2 min 100% B, 8 min: 100 % B) of eluent A (0.1% TFA in water) with eluent B (acetonitrile/water 9:1 mixture complemented with 0,1% TFA) was used. Flow rate: 1.2 mL/min. Detection: 280 and 254 nm.

Method B: Injected volume: 10 µL (supernatants derived from the stability experiment). Linear gradient elution (0 min: 0% B, 12 min: 50% B) of eluent A (0.1% TFA in water) with eluent B (acetonitrile) was used. Flow rate: 1.2 mL/min. Detection: 280 nm.

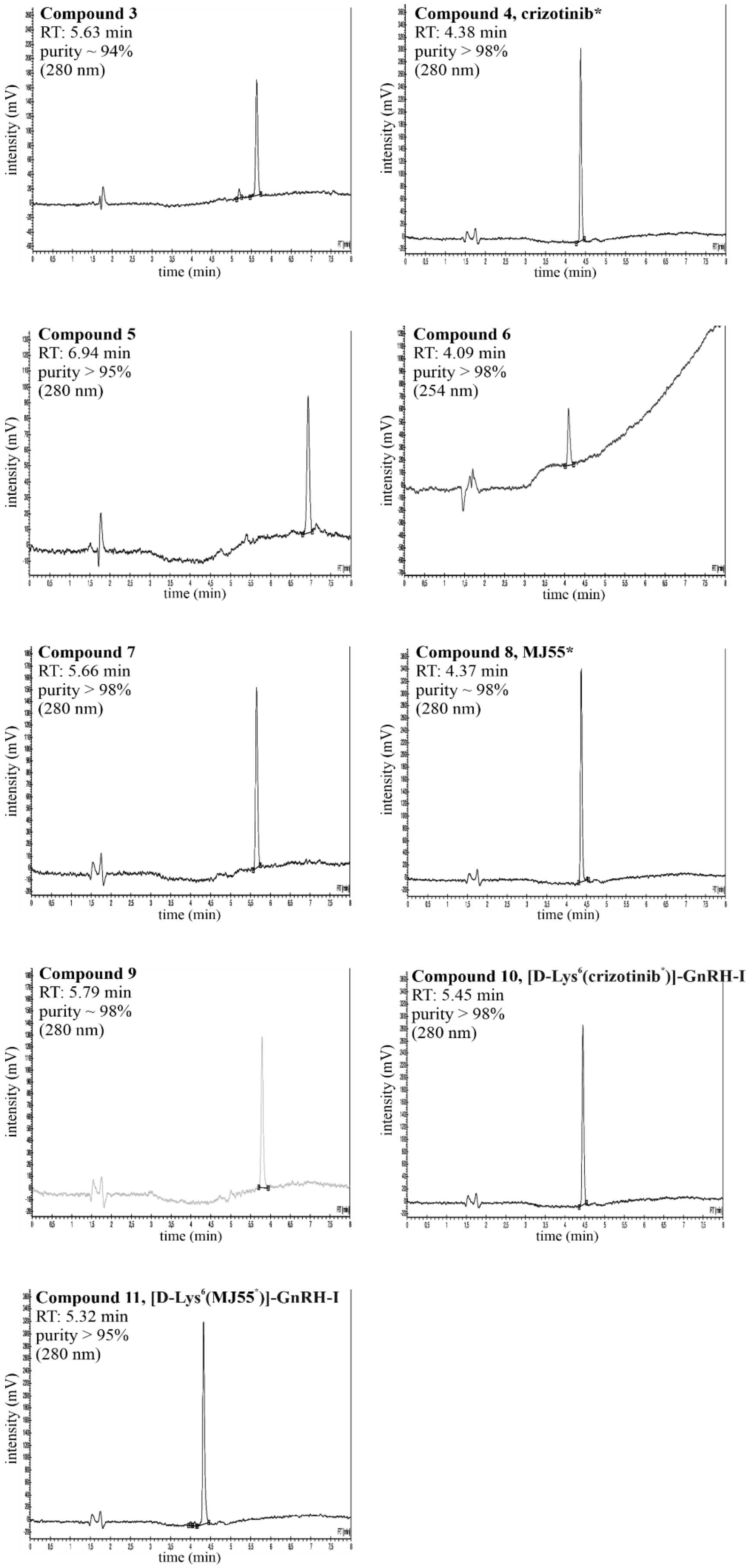

Figure S1. HPLC purity of compound 3-11. (Method A).

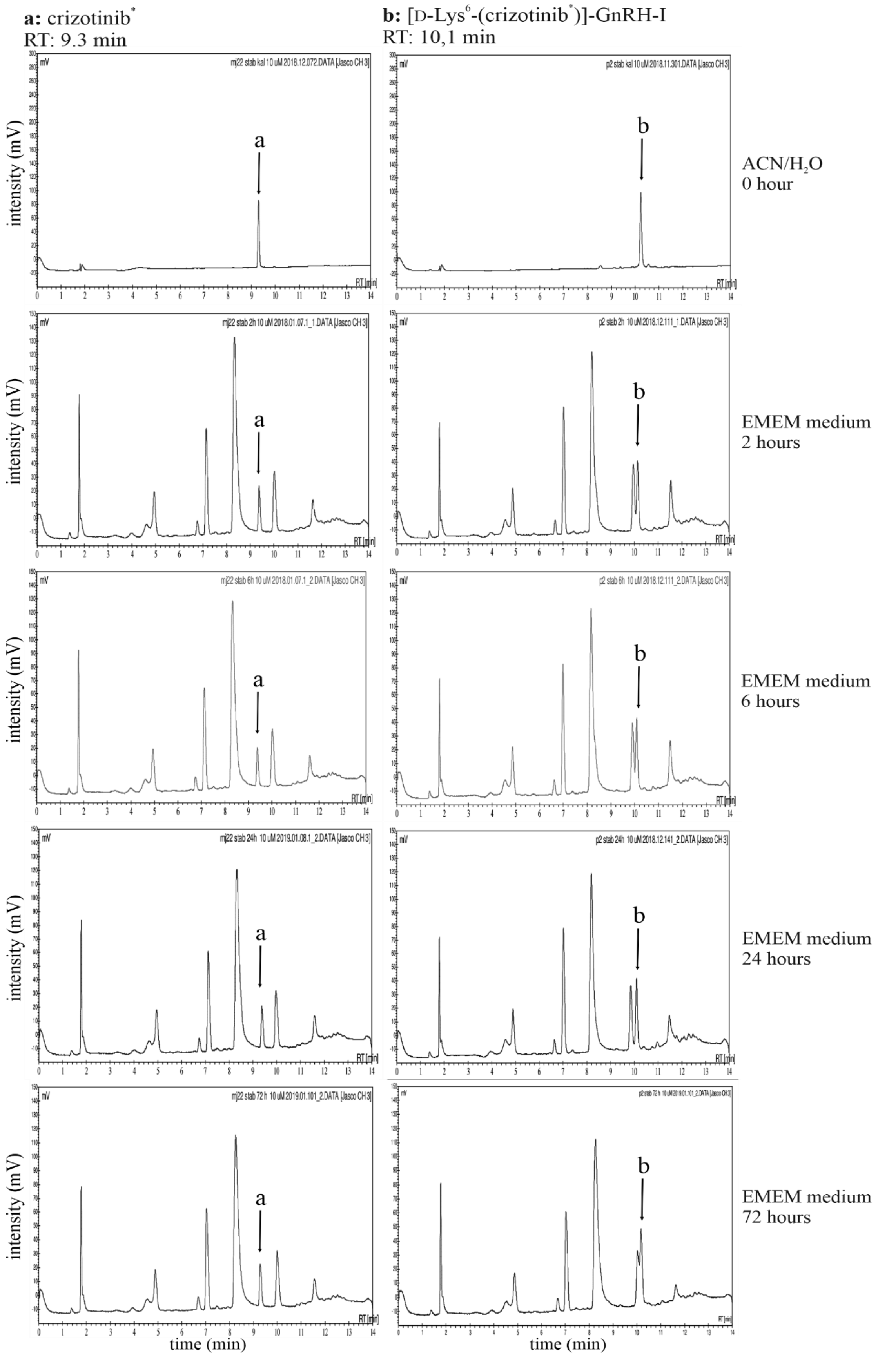

Figure S2. Stability of crizotinib* and [D-Lys6(crizotinib*)]-GnRH-I in EMEM, at 37 °C. Chromatograms were acquired by HPLC (Method B).

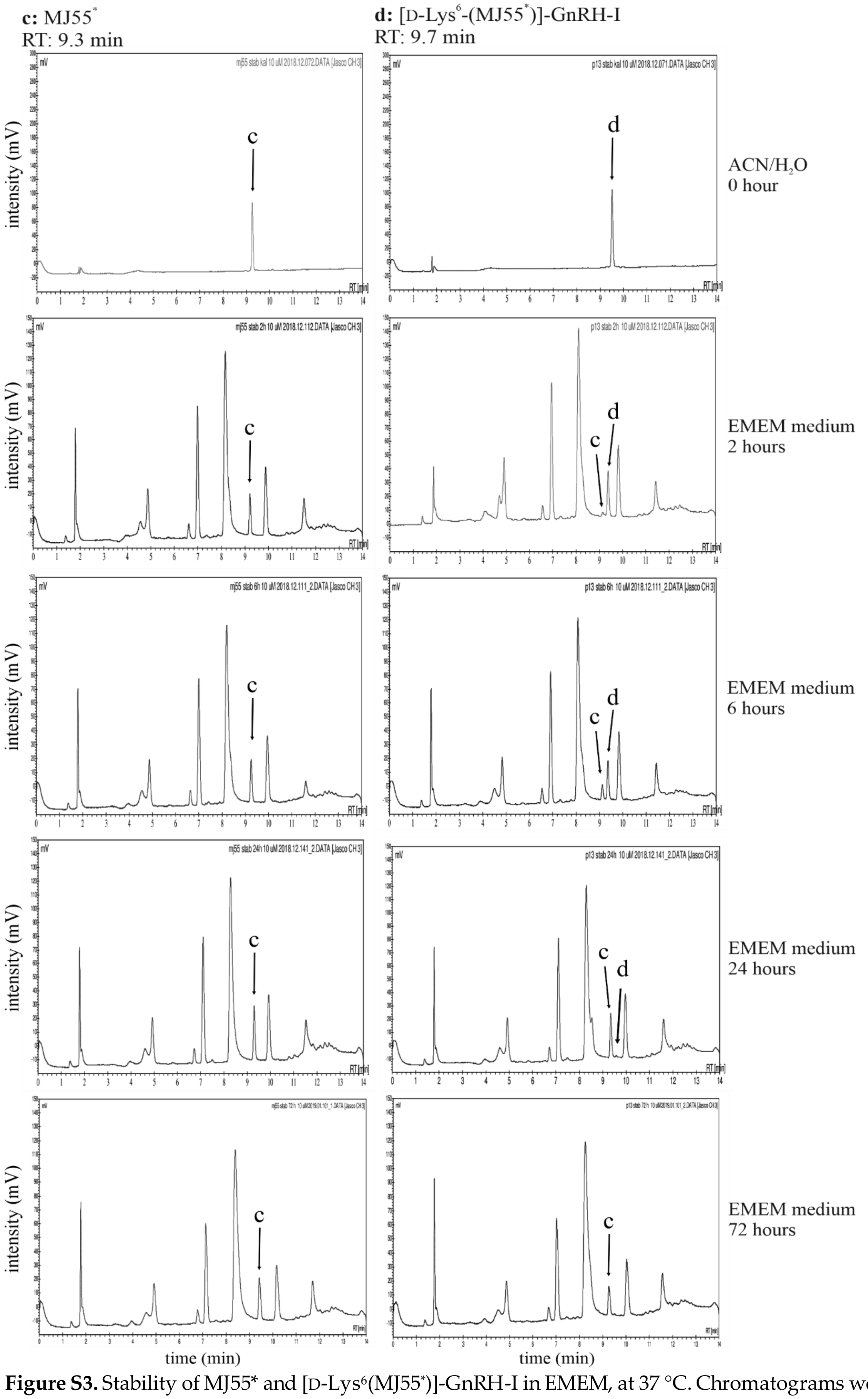

Figure S3. Stability of MJ55* and [D-Lys^6(MJ55*)]-GnRH-I in EMEM, at 37 °C. Chromatograms were acquired by HPLC (Method B).

S2.3 Structure determination of the synthetic intermediates, crizotinib and its derivatives by ^{1}H- and ^{13}C-NMR measurements

The spectra were recorded in DMSO-d_6 solution in 5 mm tubes at RT, on a Bruker DRX-500 spectrometer at 500 (^{1}H) and 125 (^{13}C) MHz, with the deuterium signal of the solvent as the lock and TMS as internal standard (^{1}H, ^{13}C). The 2D-HSQC and 2D-HMBC spectra were obtained by using the standard Bruker pulse programs.

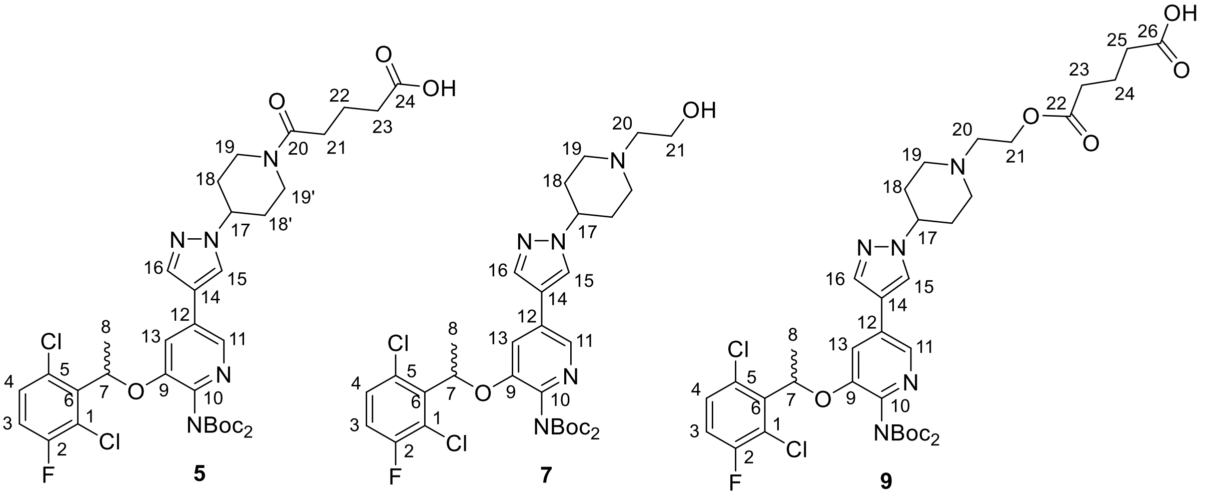

Figure S4. The appropriate numbering modes of atoms presented on the structures of **5**, **7** and **9** are used for the assignment of ^{1}H-and ^{13}C-NMR data of all the compounds characterised in the Experimental section. For compound 2 this numbering is retained starting with atom number 14 on the pyrazole ring.

The skeletal structure and the substitution pattern of the characterised compounds were unambiguously confirmed by combined use of ^{1}H- and ^{13}C-NMR methods including 2D correlation measurements such as ^{1}H-^{13}C-HSQC, ^{1}H-^{13}C-HMBC, only the following remarks are necessary to make. The presence of the *N*-substituted piperidine ring in compounds **5**-**9** was evidenced by the HMBC cross peaks generated by three-bond correlations between signal pairs H-19/C-20 and H-19′/C-20 (for **5**) and H-19/C-20 and H-20/C-19 (for **6**-**9**). Giving further support for the *N*-acylation of the piperidine unit in **5**, the separated H-19 and H-19′ signals discernible in its ^{1}H NMR spectrum are significantly downfield shifted (e.g. δH-19$_{equatorial}$/=4.49 ppm/ δH-19′$_{equatorial}$=3.94 ppm) relative to the H-19 shifts measured for **3**, **4** and **6**-**8** (δH-19$_{equatorial}$=2.8-2.9 ppm). However, in ester **9** the downfield-shifted H-19$_{equatorial}$ H-19$_{axial}$ signals (at 3.66 ppm and 3.21 ppm, resp.) point to the fast rotation of the extended chain on the piperidine ring exerting an anisotropic deshielding effect on the aforementioned protons, which – in time average – may get in the proximity of the ester group in the backfolded oxopropanoic acid terminal. Finally, for all compounds characterised in this contribution the characteristic triplet of triplets split of the sharp H-17 signal with one large coupling constant (~11 Hz) unambiguously refers to its axial position and, consequently to equatorial position of the pyrazole fragment on the rigid piperidine ring adopting well-defined chair conformation.

S3. Confocal laser scanning microscopy

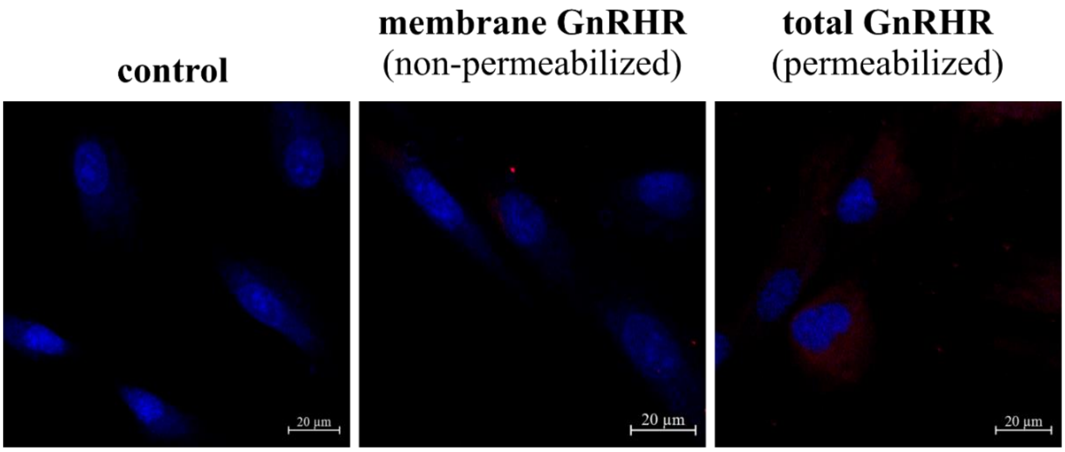

Figure S5. The GnRHR expression of human fibroblast cells is negligable, and the receptor was not detectable on the plasma membrane.

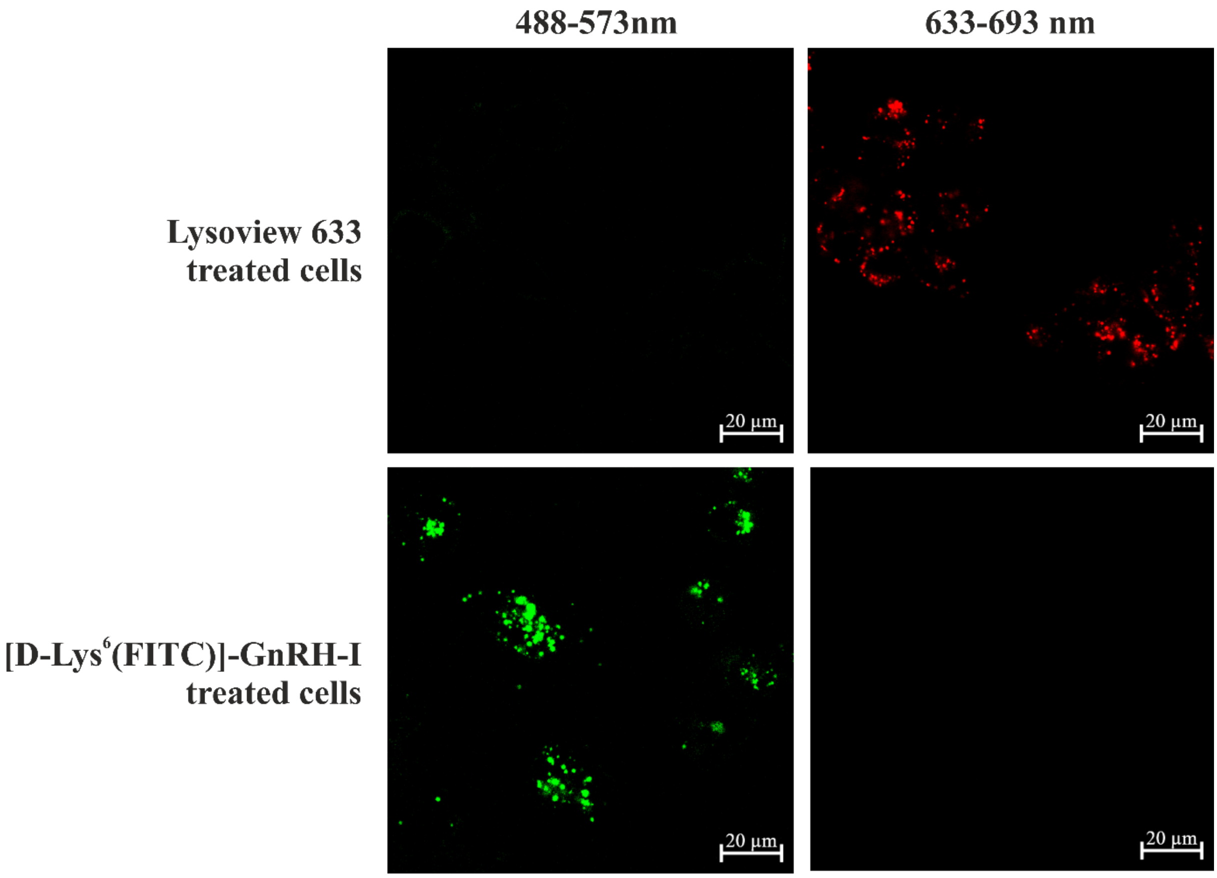

Figure S6. Confocal images of the [D-Lys^6(FITC)]-GnRH-I and the Lysoview 633 treated EBC-1 cells. No ″cross-talk″ effect was observed between the two fluorescent dye.

S4. PAMPA

Calculation formula:

$$\boldsymbol{Pe} = \frac{-\ln\left(1-\frac{Ca}{Ce}\right)}{A * \left(\frac{1}{Vd}+\frac{1}{Va}\right) * t}$$

$$\boldsymbol{Ce} = (Cd * Vd + Ca * Va)/(Vd + Va)$$

$$\boldsymbol{R} = 1 - \frac{Cd * Vd + Ca * Va}{C0 * Vd}$$

Pe = permeability (cm/s)
R = mass retention (percentage loss of compound due to non-specific binding to the plastic surfaces during the permeation assay)
A = membrane area (cm^2)
c_0 = initial donor concentration
c_a = final acceptor concentration
c_d = final donor concentration
c_e = equilibrium concentration
t = incubation time (sec)
V_a = acceptor volume (mL)
V_d = donor volume (mL)
